# Effects of Donor Rigidity on Coumarin Delayed Emission: New Tricks from Old Materials

**DOI:** 10.1021/acs.jpclett.6c00210

**Published:** 2026-04-07

**Authors:** Simon Paredis, Tom Cardeynaels, Suman Kuila, Jasper Deckers, Adrian Lathouwers, Melissa Van Landeghem, Koen Vandewal, Andrew Danos, Andrew P. Monkman, Benoît Champagne, Wouter Maes

**Affiliations:** ∇ Hasselt University, Institute for Materials Research (imo-imomec), Design & Synthesis of Organic Semiconductors (DSOS), Martelarenlaan 42, B-3500 Hasselt, Belgium; ‡ imec, imo-imomec, Wetenschapspark 1, B-3590 Diepenbeek, Belgium; § University of Namur, Laboratory of Theoretical Chemistry, Theoretical and Structural Physical Chemistry Unit, Namur Institute of Structured Matter, Rue de Bruxelles 61, 5000 Namur, Belgium; ∥ 3057 Durham University, Department of Physics, OEM Group, South Road, Durham DH1 3LE, United Kingdom; ⊥ Hasselt University, Institute for Materials Research (imo-imomec), Organic Optoelectronics (OOE), Martelarenlaan 42, B-3500 Hasselt, Belgium; # School of Physical and Chemical Sciences, 4617 Queen Mary University of London, London, E1 4NS, U.K.

## Abstract

Starting from well-studied coumarin laser dyes, a novel donor-π-acceptor emitter is designed, synthesized, and investigated using quantum chemistry and spectroscopic approaches. Altering the donor unit from a freely rotating diethylamine (Coumarin 6) or ‘rigidly planar’ julolidine (Coumarin 545) to a highly twisted phenoxazine in the newly reported material red-shifts the emission and enables thermally activated delayed fluorescence (TADF) at the cost of a reduction in photoluminescence quantum yield. Separately, unexpected room temperature phosphorescence (RTP) is observed for Coumarin 6 in film, and both coumarins show triplet–triplet annihilation (TTA) delayed emission in solution. These delayed emission properties are explained by the contrasting structural properties of the different donor moieties and especially the availability of a twisted intramolecular charge-transfer state for Coumarin 6. Ultimately the rotational freedom of the donor enables emission mechanisms that have been previously overlooked for these primarily fluorescent laser dyes, as well as TADF for the new emitter.

## Graphical abstract

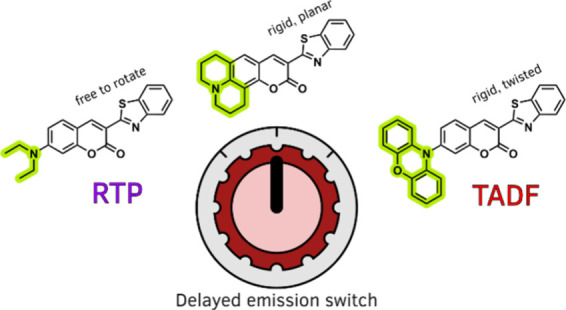

Organic light-emitting diodes (OLEDs) based on purely organic luminescent materials that are capable of harvesting triplet states have gained increasing attention due to their enhanced external quantum efficiency compared to conventional fluorescent OLEDs.^1–4^ Separate from direct triplet emission (phosphorescence; PH), triplet up-conversion to an emissive singlet state is generally achieved through either reverse intersystem crossing (rISC)^5–7^ or triplet–triplet annihilation (TTA).^8–10^ rISC followed by emission from the excited singlet state gives rise to thermally activated delayed fluorescence (TADF), and has become one of the key topics within the field of OLED research.^4,11,12^ Monoexcitonic TADF emitters have become widely prevalent because they can achieve a theoretical exciton-to-photon conversion efficiency of 100%, far exceeding the theoretical maximum of 62.5% for TTA materials which can only convert pairs of triplet excitons into individual singlet excitons.^5^

The up-conversion of triplet states in TADF materials is engineered by minimizing the singlet–triplet energy splitting (Δ*E*_*S*_1_–*T*_1__) to promote rISC.^13^ The Δ*E*_*S*_1_–*T*_1__ value can be reduced by designing materials that optimize the spatial separation of the highest occupied molecular orbital (HOMO) and the lowest unoccupied molecular orbital (LUMO). Typically, a small Δ*E*_*S*_1_–*T*_1__ is realized in highly twisted donor–acceptor (D–A) type materials with intramolecular charge-transfer (CT) excited states involving electron-rich (D) and electron-poor (A) subunits.^11,13^ Based on this design rule, hundreds of D–A TADF materials have been reported over the past few years by combining different D and A fragments, with varying performances.^4,11,14–18^ The two key features of an efficient TADF material are, therefore, rapid triplet up-conversion (*T*_1_ → *S*_1_, i.e., rISC) and efficient emissive decay from the excited singlet state (*S*_1_ → *S*_0_, i.e., fluorescence).^19,20^ However, the decoupling of HOMO and LUMO required to establish CT excited states often precludes efficient emission, due to the resulting small singlet-to-ground-state emission transition moment.^21^ As a result, molecules that prioritise rISC to the detriment of the photoluminescence quantum yield (PLQY) are frequently obtained.^22–24^

Apart from combinatorial studies of novel D or A fragments, an alternative yet largely overlooked design strategy for TADF is to start from known classes of highly fluorescent molecules and make structural adjustments to install triplet harvesting properties. Although they do not always possess CT emission, several common classes of fluorescent molecules already feature a D–A scaffold, so the proposed structural modifications to install TADF can be minimal. Some high-performance emissive materials from the coumarin family, typically employed as laser dyes, share a striking resemblance to TADF emitters we have recently reported which combine a benzothiazole (BTaz) acceptor and 10*H*-phenoxazine (PXZ) donor (Figure 1).^25,26^ In parallel, a prior investigation by Chen et al. demonstrated two TADF-active derivatives incorporating coumarin fragments that showed adequate PLQYs (±50%), emission wavelengths around 500 nm, and good performance in devices.^27^ In addition, we have recently also shown that the coumarin unit can itself also be installed to assist poorly performing TADF materials to achieve faster rISC.^28^

**Figure 1:**
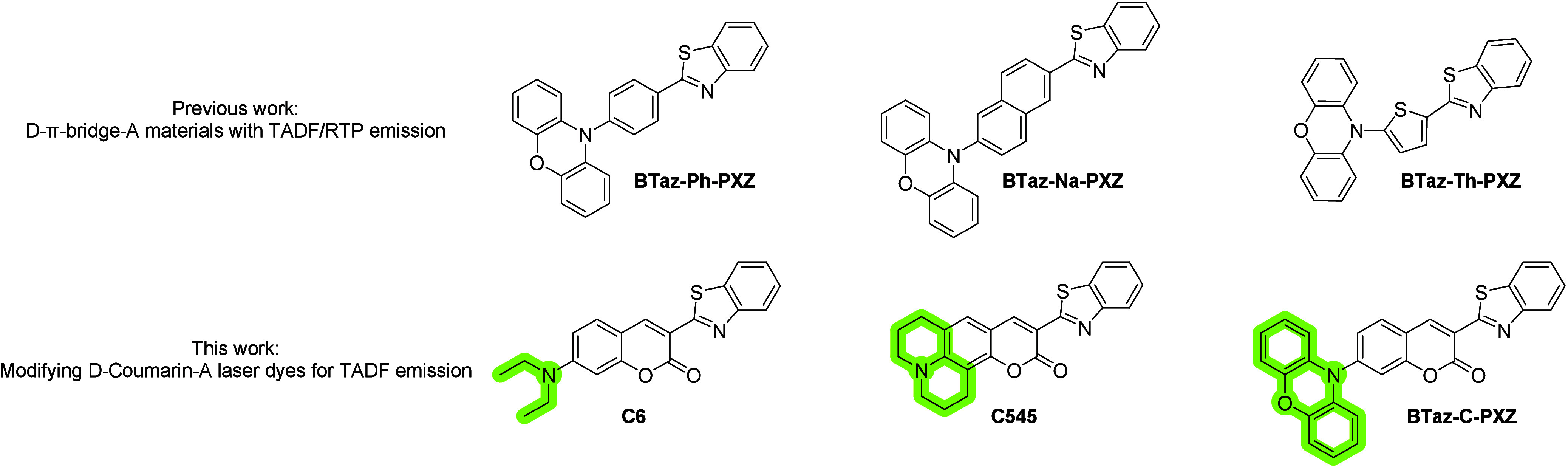
Molecular structures of **BTaz-Ph-PXZ**, **BTaz-Na-PXZ**, and **BTaz-Th-PXZ** from previous work (top)^25^ and **C6**, **C545**, and **BTaz-C-PXZ** studied here (bottom). Green highlighting emphasizes the contrasting donor groups giving rise to the different photophysical properties.

To explore the potential for installing TADF properties into these well-studied coumarin laser dyes, we have prepared the equivalent coumarin material containing a PXZ donor. As well as installing triplet harvesting properties, investigating this series of molecules allows us to compare the effects of differently structured donor groups on the excited states: the diethylamine donor in Coumarin 6 (**C6**) can rotate and potentially form twisted intramolecular charge-transfer (TICT) states,^29,30^ whereas the julolidine moiety in Coumarin 545 (**C545**) is locked ‘rigidly planar’ to the coumarin fragment of the molecule. The PXZ unit in BTaz-Coumarin-PXZ (**BTaz-C-PXZ**) is itself planar but strongly twisted with respect to the coumarin core in the ground and excited states, providing the aforementioned requirement for TADF activity. As well as engineering this TADF property in the new emitter, we also observe previously overlooked delayed emission activity in the two reference laser dyes. Computational investigation of the excited state potential energy surfaces shows that **C6**, due to its rotatable amine group, has access to relevant TICT states from which ISC and subsequent room temperature phosphorescence (RTP) arise.

The synthesis pathway for **BTaz-C-PXZ** is depicted in Scheme 1. It starts with the protection of the phenol group, whereafter a Buchwald-Hartwig cross-coupling reaction was performed to attach the phenoxazine donor. After deprotection, the final **BTaz-C-PXZ** structure was formed using a condensation reaction. Details on the synthesis procedures and characterization data are provided in the Supporting Information. **C6** and **C545** were purchased from Merck and TCI chemicals, respectively.

**Scheme 1:**
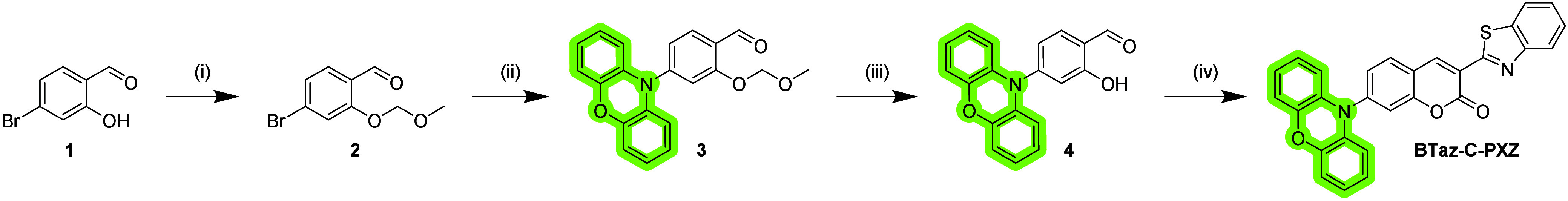
Synthesis Procedure for **BTaz-C-PXZ**: (i) *N*-Ethyl-*N*-isopropylpropan-2-amine, Chloro­(methoxy)­methane, Dichloromethane, rt, 13 h (85%); (ii) Pd­(OAc)_2_, 10*H*-Phenoxazine, XPhos, Na*t*BuO, Toluene, Reflux, 16 h (52%); (iii) HCl, MeOH, THF, 65 °C, 1 h (74%); (iv) Ethyl 2-(Benzo­[*d*]­thiazol-2-yl)­acetate, Piperidine, Ethanol, Microwave Irradiation, 20 min, 160 °C (21%)

The geometries of **C6**, **C545**, and **BTaz-C-PXZ** were fully optimized using density functional theory (DFT) calculations with M06/6-311G­(d). All geometries correspond to true minima on their potential energy surfaces with no imaginary vibrational frequencies. Time-dependent DFT (TDDFT) calculations were additionally performed to estimate the singlet and triplet energies using a modified LC-BLYP (ω = 0.17 bohr^–1^) exchange correlation (XC) functional, which is optimized for TADF research,^31,32^ within the Tamm-Dancoff approximation (TDA)^33^ and using the 6-311G­(d) basis set. TDDFT calculations were done using the polarizable continuum model (PCM) in cyclohexane to simulate a nonpolar environment. The orbital spatial distributions were calculated using the same LC-BLYP/6-311G­(d) method. All calculations were done using the Gaussian16 package.^34^ The CT character of the involved states was investigated by looking at the differences of ground and excited state electron densities. These were characterized by the distance over which the electronic charge is transferred (*d*_CT_), the amount of charge transfer (*q*_CT_), and the related change in dipole moment (Δμ), which were calculated according to the work of Le Bahers and co-workers.^35^ All the excited state calculations adopted the nonequilibrium solvation scheme.

DFT geometry optimizations show that the BTaz unit is coplanar with the coumarin part in all three molecules (Figure 2). In the case of diethyamine in **C6**, the two ethyl groups have the ability to rotate along with the unrestricted C–N bond. For **C545**, the planarized julolidine-like group is fused to the coumarin π-system, preventing rotational motion. The PXZ group of **BTaz-C-PXZ** is more restricted than the diethylamine of **C6** due to steric interactions of the large, rigid, and planar PXZ group, but has more degrees of freedom than the julolidine in **C545**. Moreover, in the ground state, the PXZ moiety is strongly twisted (i.e., 74°) with respect to the acceptor part. The HOMO and LUMO spatial distributions for both **C6** and **C545** are highly overlapping and spread over the entire molecule (Figure 2), resulting in the absence of strong CT character (for the HOMO–LUMO-based electronic transitions). Furthermore, the HOMO–1 is (mainly) located on the BTaz moiety in both cases. In contrast to the reference laser dyes, the highly twisted PXZ introduces a clear separation of the HOMO and LUMO, indicating the possibility of excited states with strong CT character.

**Figure 2:**
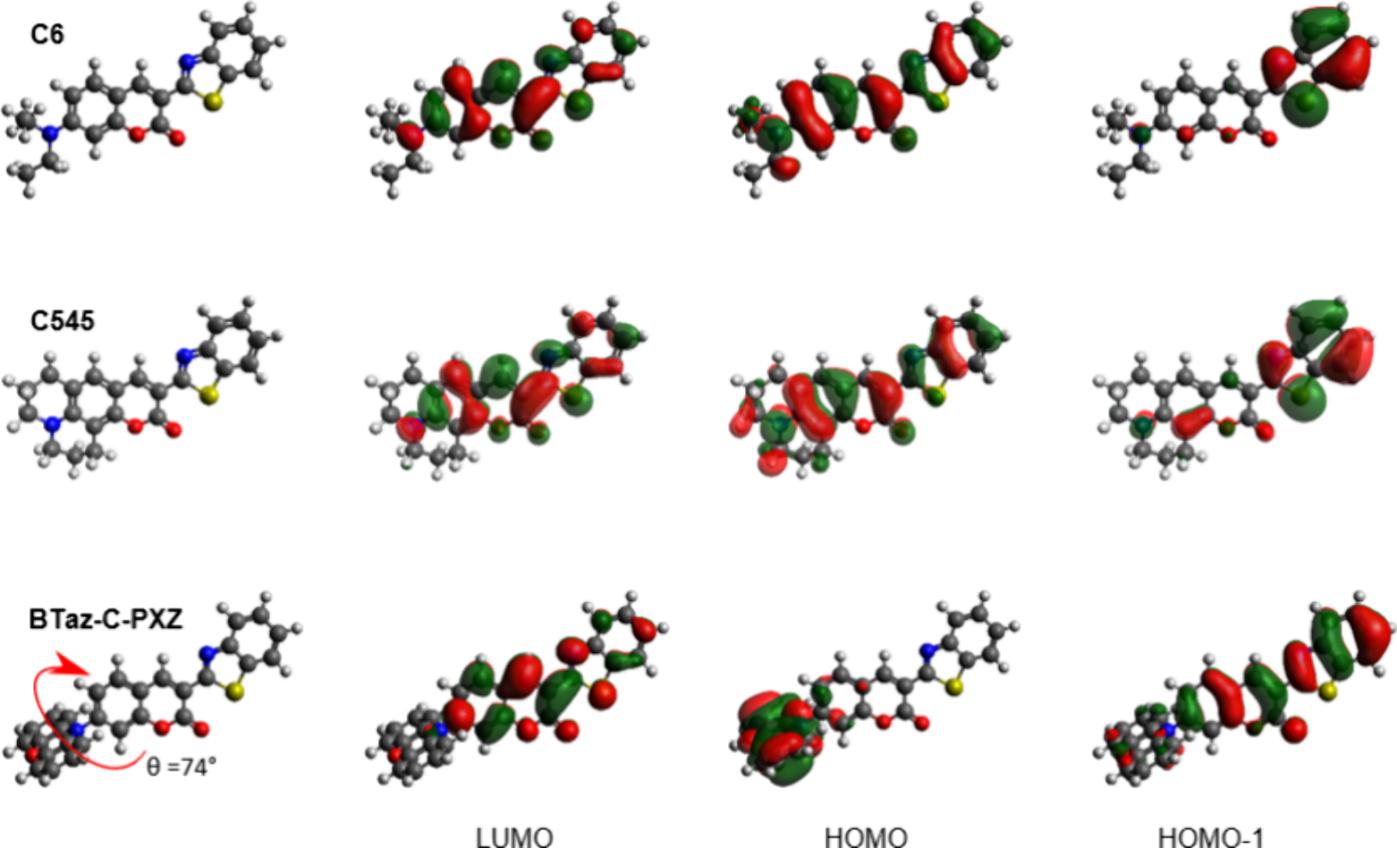
HOMO–LUMO spatial distributions for **C6**, **C545**, and **BTaz-C-PXZ**. Isocontour values of 0.02 (a.u.) were used for all orbitals.

The absence of clear HOMO–LUMO separation for **C6** and **C545** drastically affects the theoretical Δ*E*_*S*_1_–*T*_1__, calculated at 0.94 eV in both cases (Table 1). **BTaz-C-PXZ** on the other hand possesses a much smaller calculated Δ*E*_*S*_1_–*T*_1__ of 0.15 eV. In theory, this gap is small enough for triplet excitons to be up-converted to the excited singlet state via rISC, enabling TADF.^11^ Additionally, the newly designed **BTaz-C-PXZ** molecule shows a drastic decrease in the oscillator strength for the first excited singlet state (*S*_1_) compared to the known coumarin dyes. In contrast, the oscillator strength for the second excited singlet state (*S*_2_) is large. This opposite behavior arises from a switch in character from LE to CT for *S*_1_, while *S*_2_ remains localized (Table 2). The relatively high *d*_CT_, Δμ, and *q*_CT_ values for the *S*_0_ → *S*_1_, *S*_0_ → *T*_1_, and *S*_0_ → *T*_2_ transitions in Table 2 indicate a clear increase of the CT characteristics for **BTaz-C-PXZ** compared to the coumarin laser dyes. These CT characteristics are also visualized in Figure S1, detailing the difference in electron density between the ground and (different) excited states.

**Table 1:** TDDFT Results for the Vertical First and Second Singlet Excitation Energies (and Corresponding Oscillator Strengths *f*) and the First and Second Vertical Triplet Excitation Energies^a^

Compound	*S*_1_ (eV)	*f*_*S*_1__	Nature	*S*_2_ (eV)	*f*_*S*_2__	Nature	*T*_1_ (eV)	Nature	*T*_2_ (eV)	Nature	Δ*E*_*S*_1_–*T*_1__ (eV)	Δ*E*_*S*_2_–*T*_1__(eV)
**C6**	3.23	1.491	H → L	4.07	0.021	H-1 → L	2.29	H → L	3.34	H-1 → L	0.94	1.05
**C545**	3.13	1.484	H → L	3.93	0.001	H-3 → L	2.19	H → L	3.29	H-1 → L	0.94	1.10
**BTaz-C-PXZ**	2.46	0.133	H → L	3.53	0.967	H-1 → L	2.31	H → L	2.59	H-1 → L	0.15	0.28

a The dominant nature of the one-particle excitations is also given (H = HOMO, L = LUMO).

**Table 2:** Calculated Charge-Transfer Distance (*d*_CT_), Change in Dipole Moment upon Excitation (Δ*μ*, Excited State Dipole – Ground State Dipole), and the Amount of Charge Transferred (*q*_CT_) Accompanying the *S*_0_ → *S*_*n*_ and *S*_0_ → *T*_*n*_ (*n* = 1, 2) Transitions in Cyclohexane

	*S*_0_ → *S*_1_			*S*_0_ → *S*_2_			*S*_0_ → *T*_1_			*S*_0_ → *T*_2_		
Compound	*d*_CT_ (Å)	Δμ (D)	*q*_CT_ (e)	*d*_CT_ (Å)	Δμ (D)	*q*_CT_ (e)	*d*_CT_ (Å)	Δμ (D)	*q*_CT_ (e)	*d*_CT_ (Å)	Δμ (D)	*q*_CT_ (e)
**C6**	1.85	3.67	0.41	3.24	8.65	0.56	0.98	2.15	0.46	0.77	1.56	0.42
**C545**	2.11	4.16	0.41	1.50	3.45	0.48	2.26	5.17	0.48	0.46	0.91	0.41
**BTaz-C-PXZ**	4.18	22.48	1.12	0.79	1.70	0.45	3.46	13.26	0.80	1.95	5.65	0.60

Steady-state absorption and fluorescence spectra of the three coumarin emitters in toluene solution (50 μM) and in zeonex films (1 or 0.1 w/w %) are shown in Figure 3, and the most important data are gathered in Table 3. For **C6** and **C545** the absorption and emission bands in solution and in the solid state are attributed to LE singlet states due to the narrow and structured peak shape and are supported by the simulated TDDFT absorption spectra, shown in Figure S2. For **BTaz-C-PXZ** the absorption spectrum in toluene consists of two bands: an intense higher energy peak around 367 nm and a weak, broad, lower-energy peak at 468 nm, which we attribute to LE and direct CT absorption, respectively (Figure 3). Both absorption and emission maxima in **C545** are red-shifted compared to **C6**, presumably due to the stronger donating ability associated with the planarized julolidine group. Films of these laser dye materials were measured at 0.1 w/w % doping to help avoid aggregation of their flat and electron-rich structures, which is evident from strong time-dependent spectral red-shifts in time-resolved measurements of 1 w/w % films (*vide infra*, Figure S6). For **BTaz-C-PXZ** similar aggregation was not observed presumably due to the steric bulk of the twisted PXZ unit, and 1 w/w % films were used to maintain an adequate emission signal for this lower-PLQY CT-emitting material. Because of these CT excited states, the emission for **BTaz-C-PXZ** is broad and relatively unstructured, both in solution and in the solid state. Moreover, the emission in the more polar solvent toluene (λ_max_ = 630 nm) is red-shifted with respect to the lower polarity zeonex host (λ_max_ = 562 nm) due to stabilization of the CT state, in contrast to **C6** and **C545** which are only minimally impacted.

**Figure 3:**
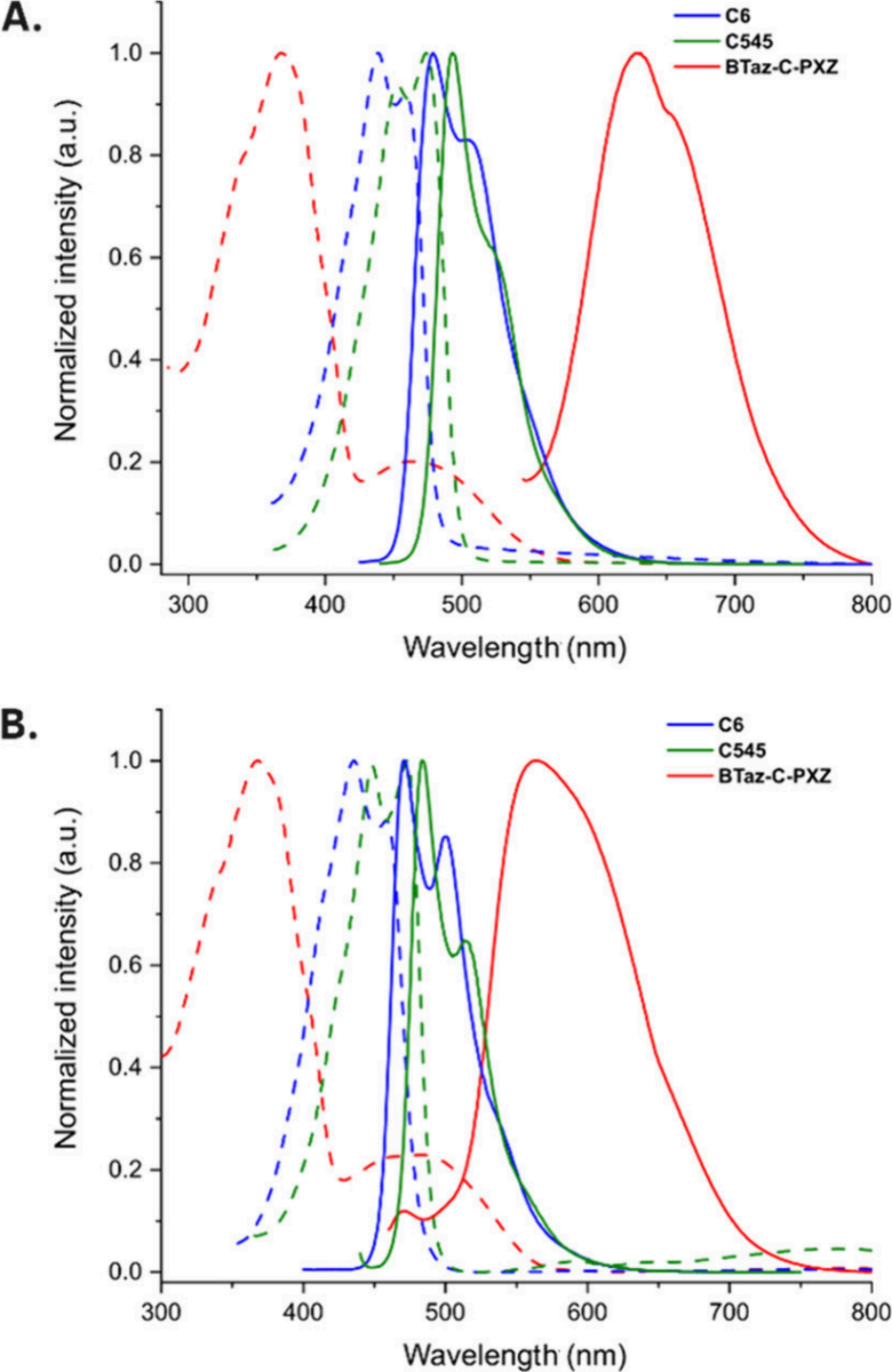
Normalized steady-state absorption (dashed lines) and emission spectra (solid lines) in toluene (A) and zeonex (B) for **C6** (blue, 0.1 w/w % film), **C545** (green, 0.1 w/w % film), and **BTaz-C-PXZ** (red, 1 w/w % film).

**Table 3:** Spectroscopic Data for the Three Coumarin-Based Molecules

Compound		λ_abs_ (nm)^a^	*ε* (× 10^4^) (M^–1^ cm^–1^)^b^	λ_em_ (nm)^c^	Φ_f,atm_ ^d^ ^,^ ^f^	Φ_f,inert_ ^e^ ^,^ ^g^	Φ_Δ_ ^h^
**C6**	Toluene	438	4.8	479, 507		0.71	∼0
	Zeonex (0.1 wt %)	435	/	460, 493	0.68	0.67	/
**C545**	Toluene	450	4.0	493, 523		0.74	∼0
	Zeonex (0.1 wt %)	448	/	472, 505	0.53	0.53	/
**BTaz-C-PXZ**	Toluene	367, 468	1.9, 0.41	630		0.02	0.24
	Zeonex (1 wt %)	366, 478	/	562	0.15	0.25	/

a Absorption maxima.

b Molar extinction coefficients at the absorption maxima.

c Fluorescence emission maxima.

d Photoluminescence quantum yields in toluene solution under normal atmosphere determined vs quinine (Φ_f_ = 0.58, λ_exc_ = 347 nm in 0.1 M H_2_SO_4_).

e Photoluminescence quantum yields in toluene solution under an inert atmosphere determined vs quinine.

f Absolute PLQYs in zeonex using an integrating sphere in air at room temperature.

g Absolute PLQYs in zeonex using an integrating sphere in an inert atmosphere at room temperature.

h Singlet oxygen quantum yields in toluene solution determined vs coronene (Φ_Δ_ = 0.90, λ_exc_ = 325 nm) by monitoring the absorbance of 1,3-DPBF at 414 nm. All values have uncertainties of 1 unit in their smallest significant figure.

The PLQYs of the three emitters in toluene and zeonex film under normal and inert atmosphere (Φ_f,atm_ and Φ_f,inert_) were determined and are shown in Table 3. The PLQYs of both **C6** and **C545** are much larger than for **BTaz-C-PXZ**, which could be explained by rapid emissive decay from the localized excited *S*_1_ state to *S*_0_ (i.e., prompt fluorescence; PF) without a significantly active competing ISC channel. The slower PF of **BTaz-C-PXZ** (Figure S3) alongside the CT excited state character and the calculated small Δ*E*_*S*_1_–*T*_1__ together indicated that it may be capable of rISC and triplet harvesting relevant to devices. Crucially, only for **BTaz-C-PXZ** does the PLQY in zeonex increase under inert atmosphere, suggesting formation of oxygen-sensitive triplet excitons that meaningfully contribute to emission when protected from quenching. This increase is not observed for **C6** and **C545**, where the observed differences between air/inert measurements are within the margin of error for the sphere-based solid PLQY measurements. An alternative technique to quantify triplet formation is the singlet oxygen formation yield, determined by energy transfer from the excited fluorophore triplet state to dissolved ground-state oxygen. For both laser dyes, no singlet oxygen formation was observed in toluene, again indicating triplet formation yields are very low. For **BTaz-C-PXZ**, on the other hand, 24% of the absorbed photons are converted to singlet oxygen (Table 3, Figure S7).

Time-resolved emission spectroscopy (TRES) experiments in 1 w/w % zeonex films and toluene (50 μM) were then performed to thoroughly examine any delayed emission properties of **BTaz-C-PXZ**. Contour maps of the normalized TRES experiments in zeonex at room temperature and at 80 K are shown in Figure 4A and 4B, with the corresponding decay plots and individual spectra below. The PF of **BTaz-C-PXZ** decreases in intensity until ∼200 ns. At room temperature, after 10 μs an intense delayed fluorescence (DF) reappears at the same wavelength as the PF, suggesting TADF. At 80 K the intense DF vanishes, thereby confirming TADF as the most likely emission mechanism. At very long delay times a slightly red-shifted band attributed to phosphorescence appears. In toluene solution (Figure S8), the decay strongly indicates a triplet upconversion mechanism with delayed CT singlet emission spanning long into the microsecond region. The emission is red-shifted with respect to that in zeonex, which is an expected response of the stabilized CT state in this more polar environment. Additional decays and contours were collected for **BTaz-C-PXZ** in 10 w/w % DPEPO films but the weak DF intensity (falling below the sensitivity of the instrument at times) precluded further investigations of this emitter in OLEDs (Figure 4C, 4D). Nonetheless, using the onsets of the steady-state fluorescence measured at room temperature and the millisecond phosphorescence at 80 K, it is possible to determine the experimental energy levels of the first singlet and triplet excited states (Table 4). Figure 4D shows that the first (CT) singlet and first triplet state of **BTaz-C-PXZ** are nearly isoenergetic (Δ*E*_*S*_1_–*T*_1__ = 0.06 eV), supporting our assignment of TADF as the emission mechanism.

**Figure 4:**
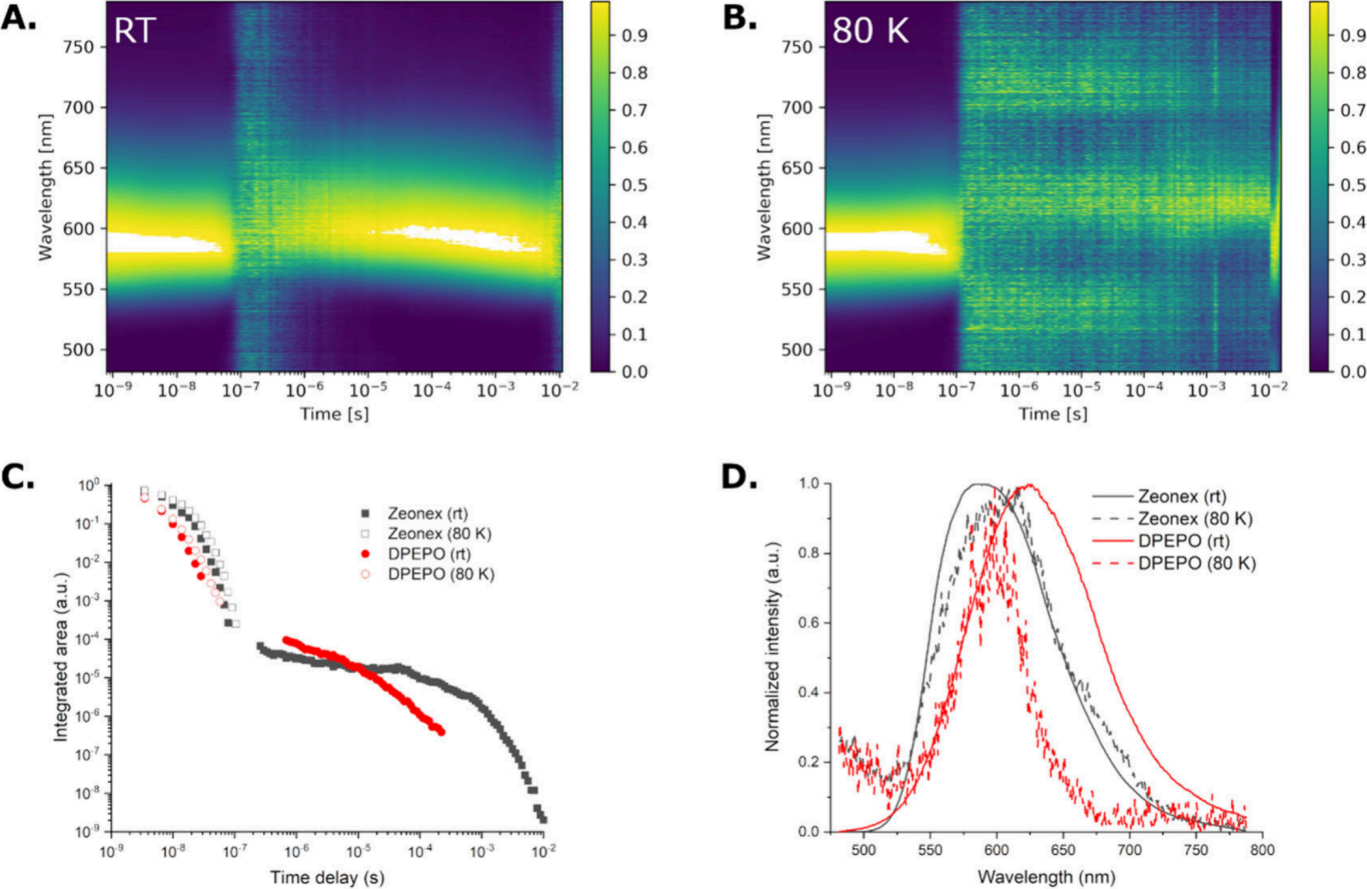
Normalized time-resolved emission spectra for **BTaz-C-PXZ** in zeonex (1 w/w %) at room temperature (A) and at 80 K (B). Decay of the total emission for **BTaz-C-PXZ** in zeonex (1 w/w %, black) and in DPEPO host (10 w/w %, red) at room temperature and at 80 K (C). Steady-state emission spectrum of **BTaz-C-PXZ** at room temperature for 1 w/w % zeonex (black) and 10 w/w % DPEPO (red) films, compared to the phosphorescence at 80 K (delay time 80 ms) (D).

**Table 4:** Singlet and Triplet Energies Derived from the Steady-State (in 0.1 w/w % Zeonex Films) and Time-Resolved Emission Spectra (in 1 w/w % Zeonex Films), Respectively

Compound	*E*_*S*_1__ (eV)^a^	*E*_*T*_1__ (eV)^b^	*E*_*T*_2__ (eV)^c^	Δ*E*_*S*_1_–*T*_1__ (eV)	Δ*E*_*T*_2_–*T*_1__ (eV)^d^
**C6**	2.74	2.62	3.34	0.12	0.72
**C545**	2.67	2.05	3.29	0.62	1.24
**BTaz-C-PXZ**	2.44	2.38	2.59	0.06	0.21

a Taken from the onset of the steady-state emission in zeonex film.

b Taken from the onset of the phosphorescence emission at ms time scales at 80 K in zeonex film. The red-shifted peak is attributed to the D–A molecule and is therefore used here.

c From calculations.

d Calculated as *E*_*T*_2__ – *E*_*T*_1__ from calculated and experimental values, respectively. All values have uncertainties of 1 unit in their smallest significant figure.

TRES experiments were then performed to examine any delayed emission properties of the reference coumarin emitters **C6** and **C545**. Contour maps of the normalized spectra for the materials in zeonex at room temperature (0.1 w/w % films) and at 80 K are shown in Figure 5, with the corresponding individual spectra in Figure S5 and decay plots shown in Figure S3. For **C545** the PF vanishes after ∼200 ns, with no subsequent delayed emission observed at room temperature. **C545** shows delayed emission in the millisecond regime at 80 K which spectrally matches the PF (Figures 5D and S5D), which we attribute to TTA as TADF is not expected for a material with such a large Δ*E*_*S*_1_–*T*_1__ and at such low temperatures. At 80 K, triplet excitons can possess a lifetime long enough for migration and TTA (which itself is not thermally promoted) to occur.^36–38^ At higher temperatures, TTA can be suppressed by more active vibrational quenching of triplet excitons. At significantly later times, an additional red-shifted PH band is also observed for the 1 w/w % film (Figures S4 and S6). In toluene solution (50 μM) (Figure S8), the TRES decay also shows a delayed component with a similar emission to the prompt fluorescence and this is also attributed to TTA due to the large Δ*E*_*S*_1_–*T*_1__.

**Figure 5:**
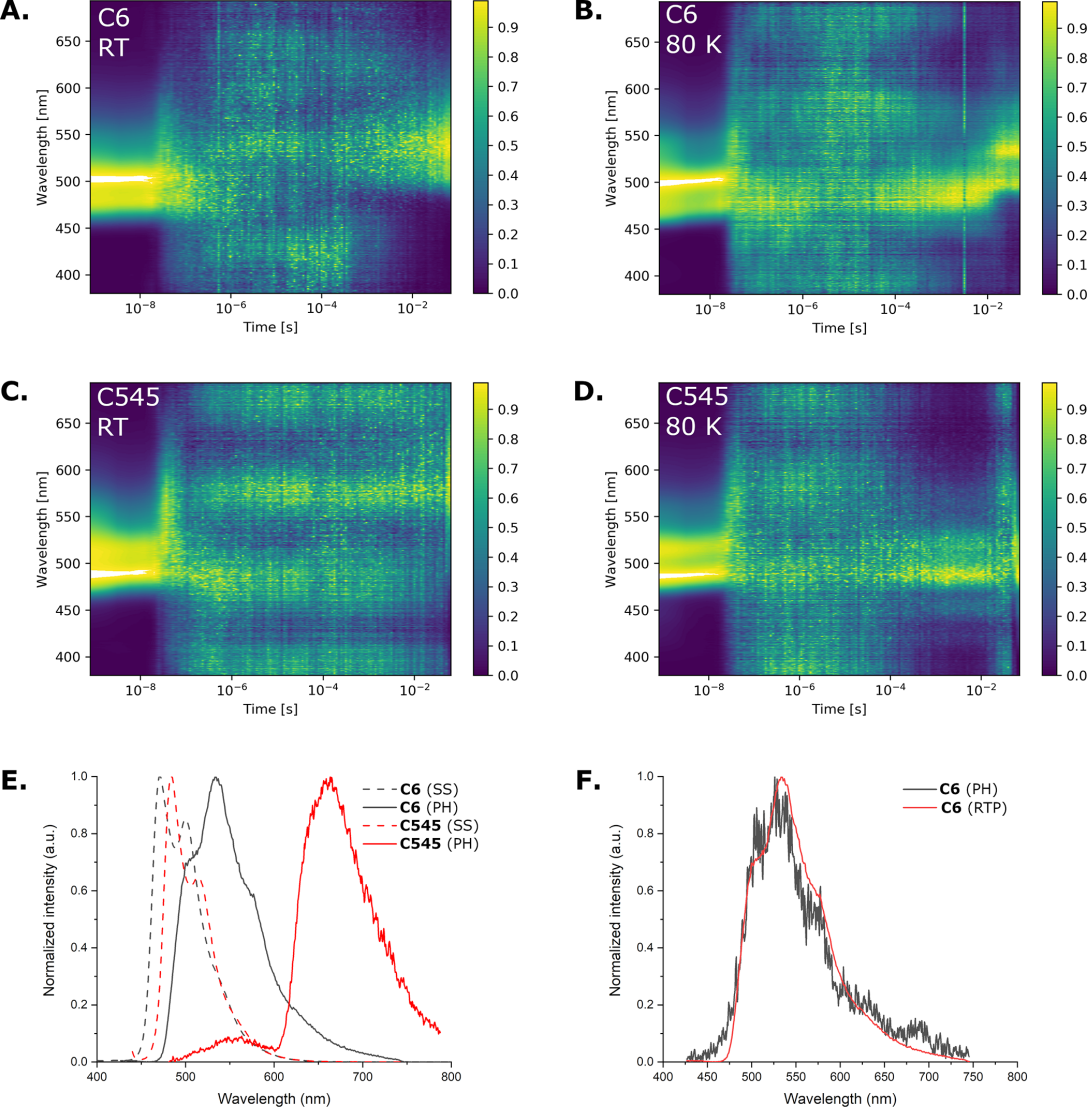
Normalized time-resolved emission spectra for **C6** (A,B) and **C545** (C,D) in zeonex (0.1 w/w %) at room temperature and at 80 K. The noise arising between the prompt and delayed emission represents the noise floor of the hardware. Steady-state PL and delayed (80 ms) PH/RTP spectra are compared in panels E and F. The feature at ∼530 nm in the **C545** PH spectrum is attributed to residual aggregate emission in this 1 w/w % film (Figure S4), as higher doping was required to acquire a phosphorescence signal for this material.

The PF of **C6** in zeonex at room temperature drops below the hardware detection limit after ∼200 ns, with emission surprisingly reappearing in the millisecond range (Figure 5A). Moreover, this millisecond emission is red-shifted with respect to the PF, suggesting an RTP emission mechanism. This assignment is confirmed by comparison to the PH spectrum observed at similar delay times at 80 K, where the emission profile of the delayed emission at room temperature clearly matches the PH spectrum (Figure 5F). As well as PH, **C6** at 80 K shows delayed emission in the early millisecond regime which is not red-shifted like the later PH emission (Figures 5B and S5B). The millisecond delayed emission of **C6** has the same wavelength as the PF, which we attribute to TTA similar to **C545**. The TRES data in toluene (50 μM) show similar behavior to those of **C545** (Figure S8).

While it is surprising to observe TTA in such diluted films, the aggregation behavior exhibited in 1 w/w % films (necessary to obtain adequate phosphorescence signal at lower temperatures) indicates that **C6** and **C545** are particularly effective at forming aggregates in these solution-processed films (Figure S6).^39^ Aggregates may therefore exist in highly concentrated clusters in these films, that form as the films are cast from solvent and then gradually dry, beyond what may be expected from the low bulk concentrations.

Using the onset of the steady-state fluorescence measured at room temperature and the onset of the millisecond PH at 80 K (Figure 5E), the Δ*E*_*S*_1_–*T*_1__ was found to be 0.12 eV for **C6** and 0.62 eV for **C545** (Table 4). While the larger value for **C545** is in line with prior expectations, having no clear HOMO–LUMO separation and the absence of CT character in the first singlet and triplet excited state, the singlet–triplet gap of **C6** is instead surprisingly small. Reports in literature on dyes containing the dimethylamino group suggest that a twisted intramolecular charge transfer (TICT) state is available for these compounds.^28,29^ In order to investigate this possibility for **C6**, additional calculations were performed.

Because the **C6** ethyl groups have many conformational degrees of freedom, potential energy surfaces (PES) were obtained for the computationally simpler dimethyl variant, targeting the C–C–N–C dihedral angle of the dimethylamino group (Figure 6). Calculations were performed using the LC-BLYP17/6-311G­(d) method (PCM with cyclohexane) for the ground state and for the *S*_1_, *T*_1_, and *T*_2_ excited states (Figures S9 and S10) across a range of forced D–A dihedral angles. While the ground-state (GS) PES shows a substantial increase in energy when going from 0° (planarized) to a 90° (perpendicular) dihedral angle, the *S*_1_ PES is overall much flatter and shows a second minimum at ±90°. The *T*_1_ PES resembles that of *S*_0_, with relaxed dihedral angles closer to 0° and an energy maximum at ∼90°. The *T*_2_ PES is nearly isoenergetic with *S*_1_ and, similarly, has a small energy barrier for dihedral rotation.

**Figure 6:**
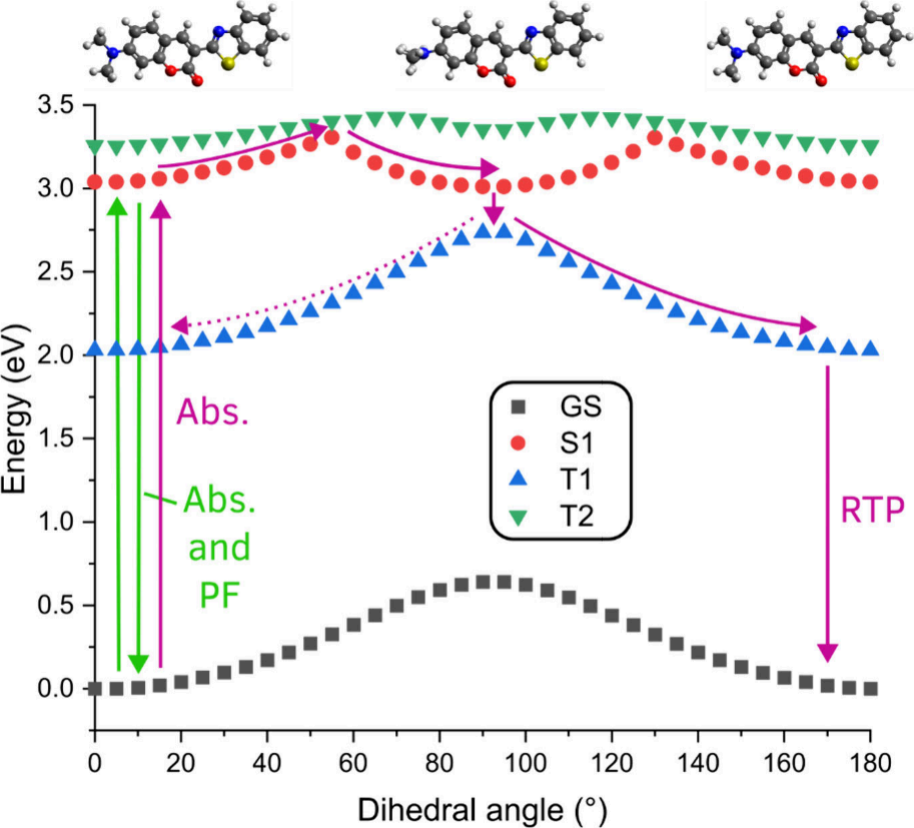
Potential energy surface scans for the methyl-substituted **C6** analogue, in which the dihedral angle was systematically altered from 90° → 0° and 90° → 180°, with increments of 5° for the ground state (GS) and 10° for the excited states. Because of an interconversion between *T*_2_ and *T*_1_ during the excited state geometry optimization of *T*_2_, the PES for *T*_2_ shown here is derived from the *T*_1_ geometry. Purple arrows show the cycle of excitation, TICT state formation, ISC, relaxation, and eventual *T*_1_ phosphorescence.

To explain the contrasting RTP activity of **C6** and inactivity of **C545**, we interpret the PES as follows: upon excitation (355 nm laser, ∼3.5 eV photons), many of the molecules in the zeonex films will absorb them and immediately emit from *S*_1_ in a nearly unchanged excited state geometry, giving rise to the experimentally observed rapid PF for both emitters. In **C6** the energy required for donor-amine rotation is surprisingly modest, with a barrier of only ∼0.25 eV separating the relaxed planar *S*_1_ energy and the alternate minimum of the perpendicular TICT state. Some molecules will overcome this barrier (Figure 6, purple horizontal arrow), with their donor rotation only mildly hindered by the semifluid zeonex environment. Having reached this TICT state which possesses considerably reduced emissivity and smaller Δ*E*_*S*_1_–*T*_1__, ISC can proceed to indirectly populate the *T*_1_ state to a greater degree than from the initially excited (planar) *S*_1_ state alone. Parallel access to *T*_1_ through direct vertical excitation to *T*_2_ (also with a small barrier to of rotation) and subsequent internal conversion may also contribute.^40^ Having reached the *T*_1_ state in its TICT configuration, the **C6** molecule will then relax to a planarized geometry again, increasing its emission oscillator strength and eventually resulting in the observed RTP emission. It is important to note though that the contribution of RTP to the total emission is still (very) small, leading to no significant difference in PLQYs in inert environments, and a near-zero singlet oxygen yield. Similar processes will be inactive in **C545**, with dual covalent C–C bond cleavage requiring an additional ∼7 eV for the same donor rotation. In fluid toluene the long process of phosphorescence is interrupted by molecular collisions, leading instead to TTA as we have previously reported for similar systems.^26^ In contrast, the first CT singlet and first triplet state of **BTaz-C-PXZ** are nearly isoenergetic (experimental Δ*E*_*S*_1_–*T*_1__ = 0.06 eV), while the LE-character *T*_2_ state is also nearby to promote vibronic coupling (theoretical Δ*E*_*T*_2_–*T*_1__ = 0.21 eV) and so rISC and TADF dominate the photophysics in this material.

In conclusion, here we demonstrate an alternative design strategy to obtain novel TADF emitters based on known and highly emissive dyes. A donor–acceptor structure was synthesized based on two common coumarin laser dyes, i.e., **C6** and **C545**, with the only difference being the nitrogen-containing donor moiety attached to the coumarin-benzothiazole acceptor unit. **C6** contains a freely rotating diethylamine and **C545** a planarized and intramolecularly locked julolidine-based donor, while the newly designed **BTaz-C-PXZ** instead contains a highly twisted phenoxazine moiety. In films and in solution, **BTaz-C-PXZ** shows exclusively CT emission and TADF delayed emission unlocked by its donor substituent, while the two laser dyes show LE emission. Furthermore, we show previously unreported delayed emission properties for the laser dyes, with **C6** displaying RTP in a 0.1 w/w % zeonex host and both **C6** and **C545** showing TTA in toluene solution. In all cases, the delayed fluorescence properties can be understood by considering the divergent structural properties of the different donor moieties, which drastically affect the complex interplay between excited state energy levels. Through its access to TICT states the triplet yield for **C6**, although objectively low, becomes large enough for RTP to be observed in contrast to **C545**.

The newly reported **BTaz-C-PXZ** ultimately displays the same trade-offs between molecular structure (planarity, rigidness and large D–A angle) and optical properties (emission wavelength, PLQY, rISC rate) that is typical for D–A TADF emitters. While the obtained PLQY and delayed emission properties are unremarkable compared to state-of-the-art TADF emitters, the design principle that this material represents is simple and underexploited. This approach may support future efforts, enabling facile synthesis and up-scaling of new materials based on a wide range of pre-existing fluorescent emitters.

## Supplementary Material



## Notes

Experimental details (materials, methods, material synthesis and NMR spectra), computational details (ground-excited state density difference plots, simulated UV–vis absorption spectra, calculations of the C6 TICT state and molecular coordinates used in the calculations), time-resolved emission spectra in zeonex film and toluene solution and data on the singlet-oxygen generation (PDF)
