# Supplementary material for: Effects of Donor Rigidity on Coumarin Delayed Emission: New Tricks from Old Materials
Source: J Phys Chem Lett. 2026 Apr 7;17(15):4389–98. doi: 10.1021/acs.jpclett.6c00210 (PMC13093650; doi:10.1021/acs.jpclett.6c00210)
Supplement: Supplementary file 1 [file jz6c00210_si_001.pdf]

Supporting information

Effects of Donor Rigidity on Coumarin Delayed Emission: New Tricks from Old Materials

Simon Paredis,[a,b,†] Tom Cardeynaels,[a,b,c,†] Suman Kuila,[d] Jasper Deckers,[a,b] Adrian Lathouwers,[a,b] Melissa Van Landeghem,[b,e] Koen Vandewal,[b,e] Andrew Danos,*[d,f] Andrew P. Monkman,[d] Benoît Champagne[c] and Wouter Maes*[a,b]

[a] Hasselt University, Institute for Materials Research (imo-imomec), Design & Synthesis of Organic Semiconductors (DSOS), Martelarenlaan 42, B-3500 Hasselt, Belgium; E-mail: wouter.maes@uhasselt.be
[b] imec, imo-imomec, Wetenschapspark 1, B-3590 Diepenbeek, Belgium
[c] University of Namur, Laboratory of Theoretical Chemistry, Theoretical and Structural Physical Chemistry Unit, Namur Institute of Structured Matter, Rue de Bruxelles 61, 5000 Namur, Belgium
[d] Durham University, Department of Physics, OEM group, South Road, Durham DH1 3LE, United Kingdom
[e] Hasselt University, Institute for Materials Research (imo-imomec), Organic Opto-Electronics (OOE), Martelarenlaan 42, B-3500 Hasselt, Belgium
[f] School of Physical and Chemical Sciences, Queen Mary University of London, London, E1 4NS, UK; E-mail: a.danos@qmul.ac.uk

[†] These authors contributed equally.

Table of contents

1. Materials and methods 2
2. Materials synthesis 4
3. Ground–excited state electron density differences 6
4. Simulated UV-Vis absorption spectra 7
5. Time-resolved emission spectra in zeonex film 8
6. Singlet-oxygen generation 11
7. Time-resolved emission spectra in toluene solution 12
8. Calculations of the TICT state of C6 12
9. NMR spectra 15
10. Coordinates of optimized geometries 19

1. Materials and methods

All reagents and chemicals were obtained from commercial sources and used without further purification. Dry solvents were obtained from an MBraun solvent purification system (MB SPS-800) equipped with alumina columns. Preparative (recycling) size exclusion chromatography (SEC) was performed on a JAI LC-9110 NEXT system equipped with JAIGEL 1H and 2H columns (eluent chloroform, flow rate 3.5 mL min^{-1}). Proton and carbon nuclear magnetic resonance (^1H and ^{13}C NMR) spectra were obtained on a Jeol NMR spectrometer operating at 400 MHz for ^1H (100 MHz for ^{13}C). Chemical shifts (δ) are given in ppm relative to $CDCl_3$ (δ = 7.26 ppm for ^1H NMR, δ = 77.16 ppm for ^{13}C NMR). Electrospray ionization - mass spectrometry (ESI-MS) was performed using an LTQ Orbitrap Velos Pro mass spectrometer (ThermoFisher Scientific) equipped with an atmospheric pressure ionization source operating in the nebulizer-assisted electrospray mode. The instrument was calibrated in the *m/z* range 220–2000 using a standard solution containing caffeine, MRFA, and Ultramark 1621. A constant spray voltage of 5 kV was used, and a nitrogen flow was applied at a dimensionless sheath gas flow rate of 7. The capillary temperature was set to 275 °C. A solvent mixture of HPLC-grade tetrahydrofuran and methanol (3:2) was used. Spectra were analyzed via Thermo Xcalibur Qual Browser software. Alternatively, mass spectra were recorded on a Bruker UltrafleXtreme™ MALDI-ToF/ToF system. Approximately 1 µL of the matrix solution (25 mg mL^{-1} *trans*-2-[3-(4-*tert*-butylphenyl)-2-methyl-2-propenylidene]malononitrile (DTCB) in $CHCl_3$) was spotted onto an MTP Anchorchip 600/384 MALDI plate. The spot was allowed to dry and 1 µL of the analyte solution (10 mg mL^{-1} in $CHCl_3$) was spotted on top of the matrix.

Electronic absorption spectra of the small molecule chromophores in solution were recorded on a Varian Cary 5000 UV-Vis-NIR spectrophotometer from Agilent Technologies. Corrected steady-state excitation and emission spectra of the chromophore solutions were recorded on a Horiba-Jobin Yvon Fluorolog-3 spectrofluorometer equipped with a 450 W Xe lamp as the light source, with an excitation wavelength (λ_{exc}) depending on the fluorescence quantum yield standard used and a slit width of 2 nm. Freshly prepared samples in 1 cm quartz cells were used to perform all UV-Vis-NIR absorption and fluorescence measurements. The fluorescence measurements were done under a right-angle arrangement. The standard uncertainty (square root of the variance) on the absorption and emission maxima is approximately 1 nm. Spectroscopic measurements under normal atmosphere were done in non-degassed spectroscopic grade solvents at 20 °C. An inert atmosphere was created by three consecutive freeze-pump-thaw cycles.

To determine the relative fluorescence quantum yields (Φ_f) in toluene, dilute solutions with an absorbance around 0.1 at the excitation wavelength were used. Quinine (λ_{exc} = 347 nm, Φ_f = 0.58 in 0.1 M H_2SO_4 solution) was used as a standard to determine the fluorescence quantum yields.[1] The fluorescence quantum yield of the tested compound (Φ_x) was calculated using Equation (1), in which Φ_{st} is the fluorescence quantum yield of the standard, F_x and F_{st} are the integrated fluorescence of the test compound and the standard, A_x and A_{st} are the absorbance of the test compound and the standard at

1. Brouwer A. M. Standards for photoluminescence quantum yield measurements in solution (IUPAC Technical Report). *Pure Appl. Chem.*, **2011**, *83*, 2213–2228.

the excitation wavelength, and n_x and n_{st} are the refractive indices of the solvents in which the test compound and the standard were dissolved, respectively.

$$\Phi_{f,x} = \Phi_{f,st}\frac{F_x(1-10^{-A_{st}})n_x^2}{F_{st}(1-10^{-A_x})n_{st}^2} \quad (1)$$

1,3-Diphenylisobenzofuran (1,3-DPBF) was used as a singlet oxygen ($^1O_2$) scavenger to determine the singlet oxygen quantum yields (Φ_Δ). The $^1O_2$ production was determined by monitoring the absorbance of 1,3-DPBF at 414 nm upon excitation of the respective chromophore at 325 nm using a single LED325W2 from Thorlabs (λ_{exc} = 325±5 nm, fwhm = 11 nm, *P* = 1.7 mW). To determine Φ_Δ, a relative method was used according to Equation (2). Here, *x* and *st* represent the sample and the standard, while *Φ*, *A*, *m*, and *n* represent the singlet oxygen quantum yield, the absorbance at the excitation wavelength (λ_{exc} = 325 nm), the slope of the decrease in absorbance of 1,3-DPBF over time, and the refractive index of the solvent used for the measurement, respectively. Optically matched solutions with an absorbance around 0.6 at 414 nm and 0.3 at 325 nm were used. Coronene was used as the standard (Φ_Δ = 0.90 in spectrograde toluene).[2] The solutions were continuously stirred during all measurements using a Cimarec magnetic stirrer.

$$\Phi_{\Delta,x} = \Phi_{\Delta,st}\left(\frac{1-10^{-A_{st}}}{1-10^{-A_x}}\right)\left(\frac{m_x}{m_{st}}\right)\left(\frac{n_x}{n_{st}}\right)^2 \quad (2)$$

Molar extinction coefficients were obtained by dissolving ca. 1 mg of the respective molecule in toluene (*ca.* 2 mL). This stock solution was serially diluted to yield different dye concentrations from which absorption spectra were obtained in quartz cells with 1 cm optical path length (*l*). Minimal three solutions with an absorbance between 0.01 and 0.1 at λ_{abs}(max) were used to plot the absorbance (*A*) versus molar concentration (*c*). The molar extinction coefficient (*ε*) was then determined using the Beer-Lambert law as given in Equation (3).

$$A = \varepsilon l c \quad (3)$$

Zeonex films were prepared via drop-casting using a mixture of the emitter and host (zeonex) in toluene at 1 w/w%. The initial solution concentrations were 100 mg mL^{-1} of zeonex and 1 mg mL^{-1} of emissive material, combined in equal 75 µL volumes. The films were drop-casted onto a ~1cm diameter quartz substrate at 65 °C to facilitate solvent evaporation. DPEPO films were similarly prepared using 15 µL of dye solution, and 135 µL of a 1 mg mL^{-1} stock solution of the host material in toluene. Absorption and emission spectra of the films were collected using a Varian Cary 5000 UV-Vis-NIR spectrophotometer from Agilent Technologies and a Horiba-Jobin Yvon Fluorolog-3 spectrofluorometer equipped with a 450 W Xe lamp as the light source, respectively. Time-resolved photoluminescence spectra and decays were recorded using a nanosecond gated spectrograph-coupled iCCD (Stanford) using an Nd:YAG laser emitting at 355 nm (EKSPLA) under vacuum for films. Time-resolved photoluminescence (TRPL) experiments in toluene solution were performed under excitation with a 405 nm pulsed diode laser (Thorlabs, NPL41C, 129 ns pulse length and 128 nJ per pulse). The solutions were prepared following freeze-pump-thaw cycles. Backscattered PL emission was diffracted in a grating spectrograph (Andor, Kymera 328i-D2-SIL) and registered by an intensified CCD detector (Andor, Istar DH320T-18F-74) which

2. F. Wilkinson, W. P. Helman and A. B. Ross, *J. Phys. Chem. Ref. Data*, 1993, **22**, 113–262.

allows for the time-gated acquisition of PL signals in a time range of ns up to 100's of ms. Unwanted laser reflections were eliminated from the spectrum by means of a 405 nm notch filter in front of the spectrograph entrance. All spectra were corrected for the spectral response of the set-up determined using a calibrated light source (Avantes, AvaLight-HAL-CAL-Mini).

2. Materials synthesis

*4-bromo-2-(methoxymethoxy)benzaldehyde (**2**)*

To a stirred solution of 4-bromo-2-hydroxybenzaldehyde (0.509 g, 2.53 mmol) in dry CH_2Cl_2 (11.5 mL), *N*-ethyl-*N*-isopropylpropan-2-amine (1.30 mL, 7.46 mmol) and chloro(methoxy)methane (0.3 mL, 3.95 mmol) were added at 0 °C. The mixture was stirred at room temperature for 13 h, diluted with saturated aqueous NH_4Cl (15 mL), and extracted with CH_2Cl_2 (3 × 15 mL). The combined extracts were dried ($MgSO_4$), filtered, and concentrated. The residual solid was purified by column chromatography (CH_2Cl_2/hexane 65/35) to afford 4-bromo-2-(methoxymethoxy)benzaldehyde (0.528 g, 85%) as a yellow-orange solid. ^1H NMR (400 MHz, $CDCl_3$): δ = 10.42 (d, *J* = 0.7 Hz, 1H), 7.70 (d, *J* = 8.3 Hz, 1H), 7.43 (d, *J* = 1.7 Hz, 1H), 7.24 (ddd, *J* = 8.3, 1.7, 0.8 Hz, 1H), 5.30 (s, 2H), 3.53 (s, 3H). ^{13}C NMR (100 MHz, $CDCl_3$): δ = 188.9, 159.9, 130.6, 129.6, 125.5, 124.3, 118.7, 94.9, 56.8. MS (ESI+) calcd. for $C_9H_9BrO_3$ $[M+Na]^{+\cdot}$: *m/z* 266.9632 (100%), found: 266.9639.

*2-(methoxymethoxy)-4-(10H-phenoxazin-10-yl)benzaldehyde (**3**)*

4-Bromo-2-(methoxymethoxy)benzaldehyde (**2**) (0.182 g, 743 µmol), 10*H*-phenoxazine (0.163 g, 891 µmol), palladium acetate (18.0 mg, 80.2 µmol), XPhos (74.7 mg, 157 µmol), and sodium *tert*-butoxide (0.152 g, 1.58 mmol) were dissolved in dry toluene (12 mL) under argon atmosphere. The mixture was heated to reflux for 16 h while stirring, then cooled down to room temperature, and concentrated under reduced pressure. The crude product was purified by column chromatography (silica) with CH_2Cl_2/petroleum ether (v/v = 65/35) as the eluent and a yellow solid was obtained (0.134 g, 52%). ^1H NMR (400 MHz, $CDCl_3$): δ = 10.54 (d, *J* = 0.8 Hz, 1H), 8.06 (d, *J* = 8.2 Hz, 1H), 7.25 (d, *J* = 1.8 Hz, 1H), 7.09 (ddd, *J* = 8.2, 1.8, 0.8 Hz, 1H), 6.77 – 6.60 (m, 6H), 6.07 – 6.00 (m, 2H), 5.30 (s, 2H), 3.53 (s, 3H). ^{13}C NMR (100 MHz, $CDCl_3$): δ = 188.9, 161.7, 146.2, 144.2, 133.5, 131.0, 125.3, 124.0, 123.5, 122.1, 117.4, 115.9, 113.6, 95.1, 56.8. MS (MALDI-ToF) calcd. for $C_{21}H_{17}NO_4$ $[M+H]^+$: *m/z* 347.12, found: 347.10.

*2-hydroxy-4-(10H-phenoxazin-10-yl)benzaldehyde (**4**)*

2-(Methoxymethoxy)-4-(10*H*-phenoxazin-10-yl)benzaldehyde (**3**) (0.134 g, 386.3 µmol) was dissolved in a methanol/THF mixture (v/v = 70/30; 14 mL), heated to 65 °C, and conc. HCl (37%, 5 drops) was added. After stirring for 1 h, water (20 mL) was added and the aqueous layer was extracted with ethyl acetate. The combined organic layers were washed with brine, dried over $MgSO_4$, filtered, and concentrated under reduced pressure. The final product was obtained as a yellow solid (0.086 mg, 74%) after column chromatography (silica) with CH_2Cl_2/petroleum ether (v/v = 65/35). Full conversion and

purity of **4** was confirmed by thin-layer chromatography (TLC) and ^{1}H NMR, and the product was then used as such for the following step. ^{1}H NMR (400 MHz, $CDCl_3$): 11.22 (s, 1H), 9.95 (s, 1H), 7.79 – 7.72 (m, 1H), 7.05 – 6.98 (m, 2H), 6.80 – 6.64 (m, 6H), 6.21 – 6.14 (m, 2H).

*3-(benzo[d]thiazol-2-yl)-7-(10H-phenoxazin-10-yl)-2H-chromen-2-one (**BTaz-C-PXZ**)*

A mixture of 2-hydroxy-4-(10*H*-phenoxazin-10-yl)benzaldehyde (**4**) (0.270 g, 890 µmol) and ethyl 2-(benzo[*d*]thiazol-2-yl)acetate (197 mg, 890 µmol) was dissolved in ethanol (8 mL), after which two drops of piperidine were added. The mixture was left to stir under microwave irradiation at 160 °C for 20 min. Thereafter, liquids were evaporated under reduced pressure and the crude product was purified by flash column chromatography (silica) with CH_2Cl_2/EtOAc (v/v = 90/10) as the eluent. **BTaz-C-PXZ** was further purified using preparative (recycling) SEC and obtained as an orange solid (0.084 g, 21%). ^{1}H NMR (400 MHz, $CDCl_3$): 9.13 (s, 1H), 8.12 (d, *J* = 8.2 Hz, 1H), 8.01 (d, *J* = 8.0 Hz, 1H), 7.92 (d, *J* = 8.2 Hz, 1H), 7.59 – 7.53 (m, 1H), 7.50 – 7.43 (m, 2H), 7.40 (dd, *J* = 8.3, 2.0 Hz, 1H), 6.84 – 6.73 (m, 4H), 6.71 – 6.66 (m, 2H), 6.15 (dd, *J* = 7.9, 1.4 Hz, 2H). ^{13}C NMR (100 MHz, $CDCl_3$): δ = 159.5, 159.4, 155.4, 152.5, 144.6, 144.3, 140.4, 136.9, 133.1, 131.7, 126.71, 126.68, 123.4, 123.0, 122.7, 121.9, 120.6, 118.5, 118.2, 116.1, 114.2. MS (MALDI-ToF) calcd. for $C_{28}H_{16}N_2O_3S$ $[M+H]^+$: *m/z* 460.09, found: 460.10.

3. Ground–excited state electron density differences

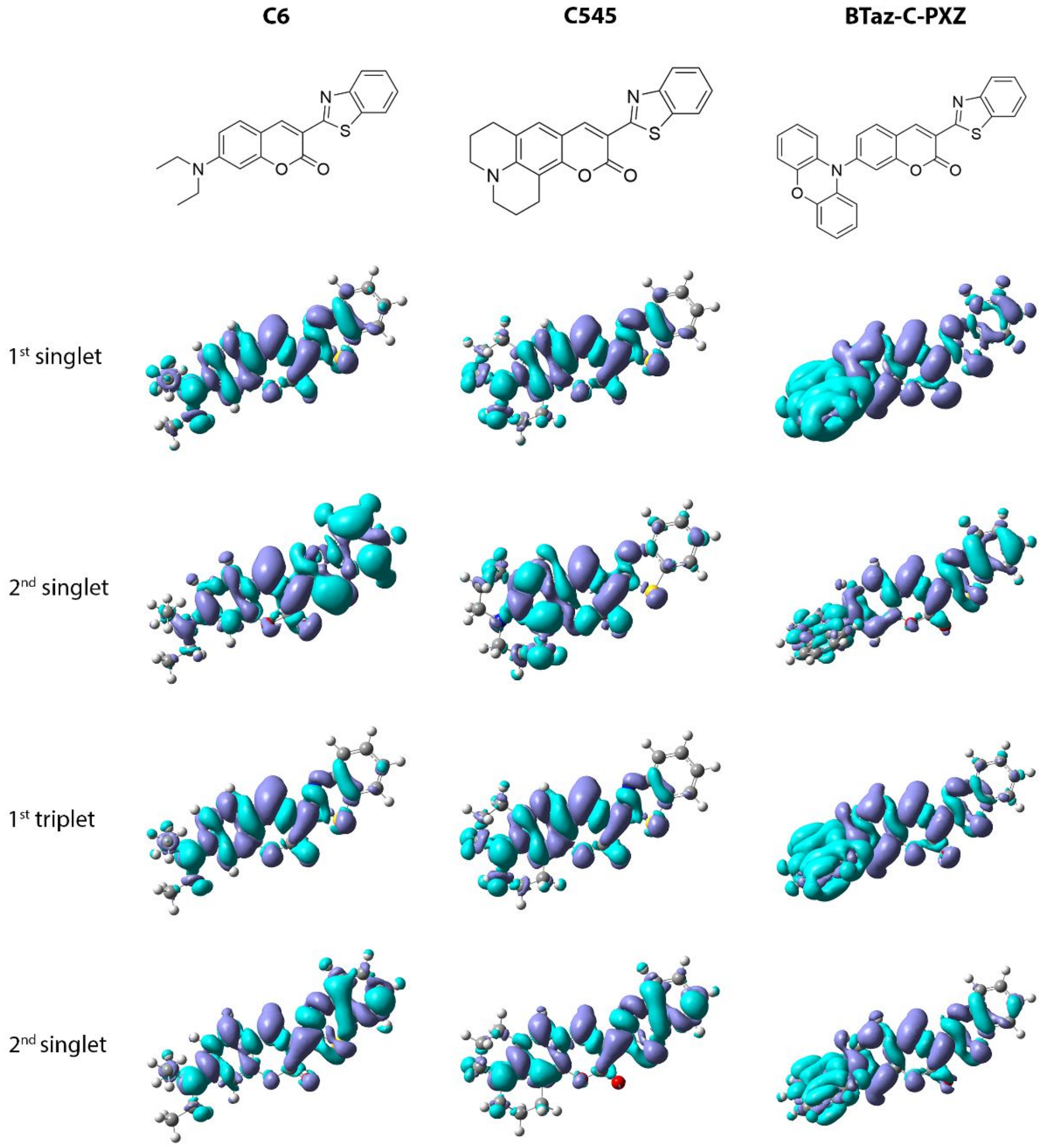

Figure S1: *Ground–excited state electron density differences for the singlet and triplet excited states of **C6** (left column), **C545** (middle), and **BTaz-C-PXZ** (right) as calculated at the PCM(cyclohexane)/TDA/LC-BLYP(ω=0.17)/6-311G(d) level of approximation. Purple areas indicate increased electron density, while cyan areas point to decreased electron density (isosurface value = 0.0004 a.u. for all densities).*

4. Simulated UV-Vis absorption spectra

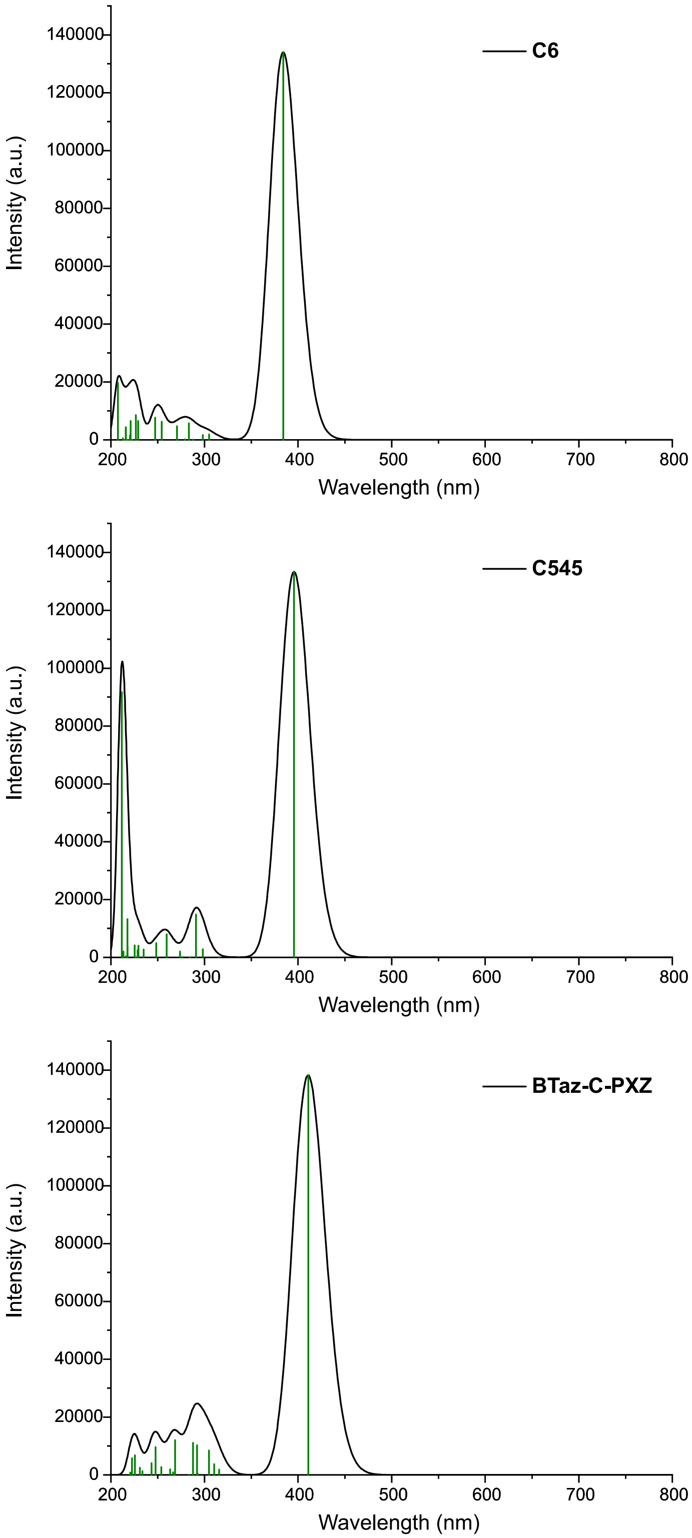

Figure S2: *Simulated UV-Vis absorption spectra derived from PCM(cyclohexane)/TDA/LC-BLYP(ω=0.17)/6-311G(d) calculations. A Gaussian function (FWHM = 0.3 eV) was associated with each excitation. It is centered on the vertical excitation energy and its intensity is proportional to the corresponding oscillator strength.*

5. Time-resolved emission spectra in zeonex film

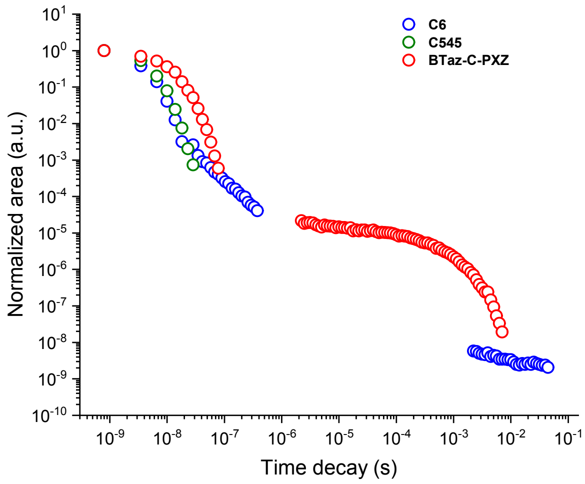

Figure S3: *Decay of the total emission for **C6** (blue, 0.1 w/w%), **C545** (green, 0.1 w/w%), and **BTaz-C-PXZ** (red, 1 w/w%) in zeonex at room temperature. Data points without signal have been omitted from the decay.*

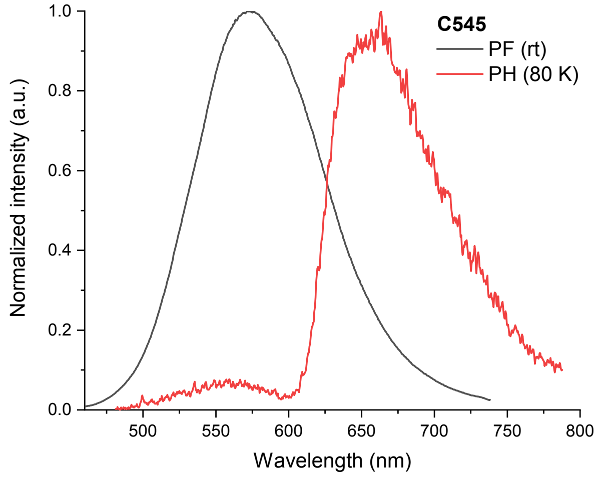

Figure S4: *Normalized prompt fluorescence (black) and phosphorescence (red) spectra for **C545** in a 1 w/w% zeonex film.*

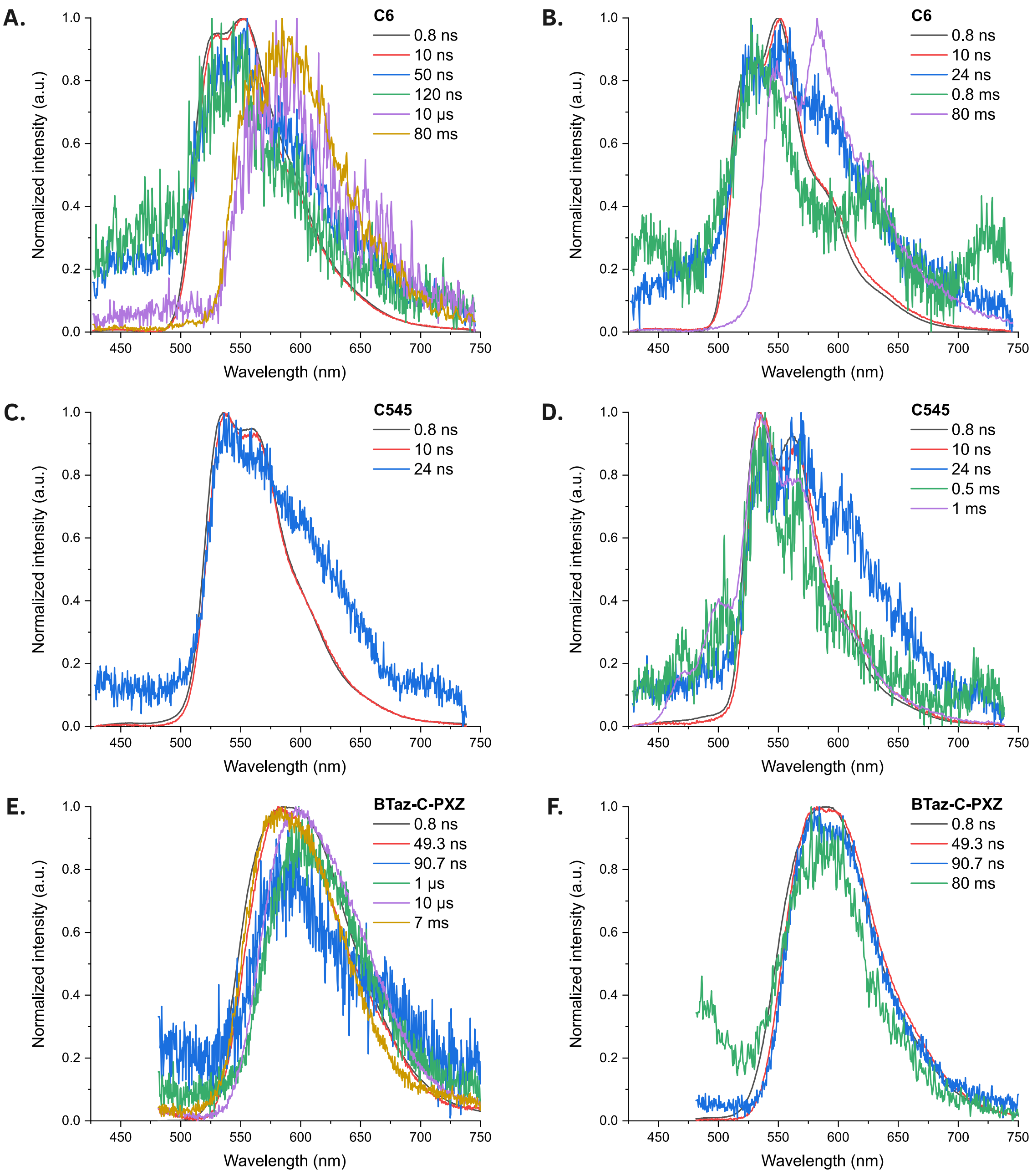

Figure S5: *Normalized time-resolved emission spectra in zeonex for* ***C6*** *(A and B; 0.1 w/w%),* ***C545*** *(C and D; 0.1 w/w%), and* ***BTaz-C-PXZ*** *(E and F; 1 w/w%) at room temperature (left) and at 80 K (right).*

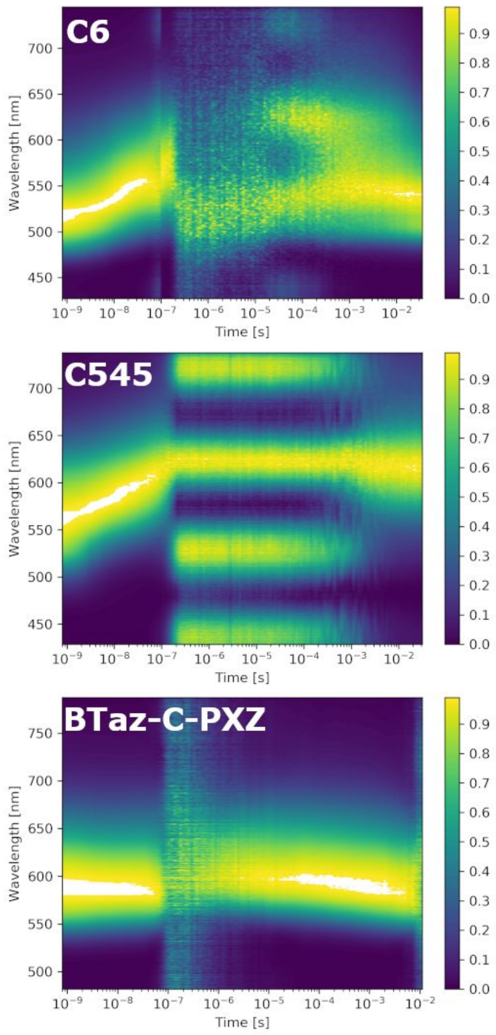

Figure S6: *Contour plots of the normalized time-resolved emission spectra for* ***C6*** *(top),* ***C545*** *(middle), and* ***BTaz-C-PXZ*** *(bottom) in a 1 w/w% zeonex film at room temperature. The red-shift of the prompt emission over time (for* ***C6*** *and* ***C545****) is indicative of dimer or aggregate emission, which is red-shifted with respect to the monomer emission observed in solution and dilute films. The noise arising between the prompt and delayed emission can be attributed to the baseline sensitivity of the iCCD camera.*

6. Singlet-oxygen generation

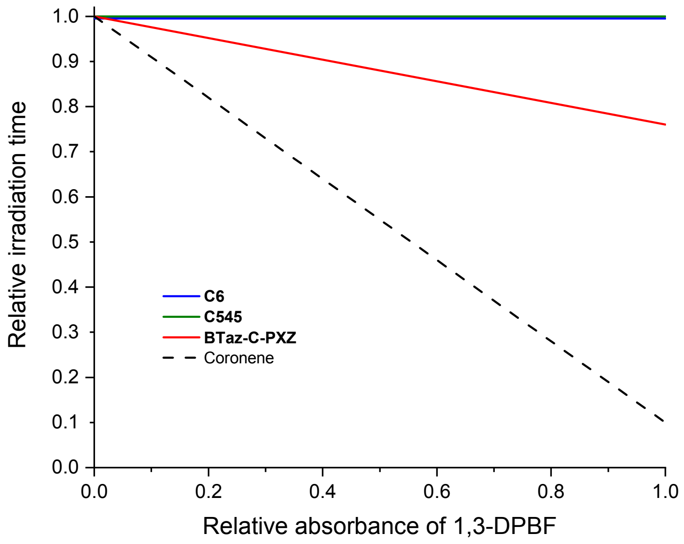

Figure S7: *Relative decrease in absorbance of 1,3-DPBF at 414 nm under continuous irradiation using a single 325 nm LED in the presence of the respective coumarin dye in toluene. Coronene was used as a standard (Φ_Δ = 0.90 in toluene).*

7. Time-resolved emission spectra in toluene solution

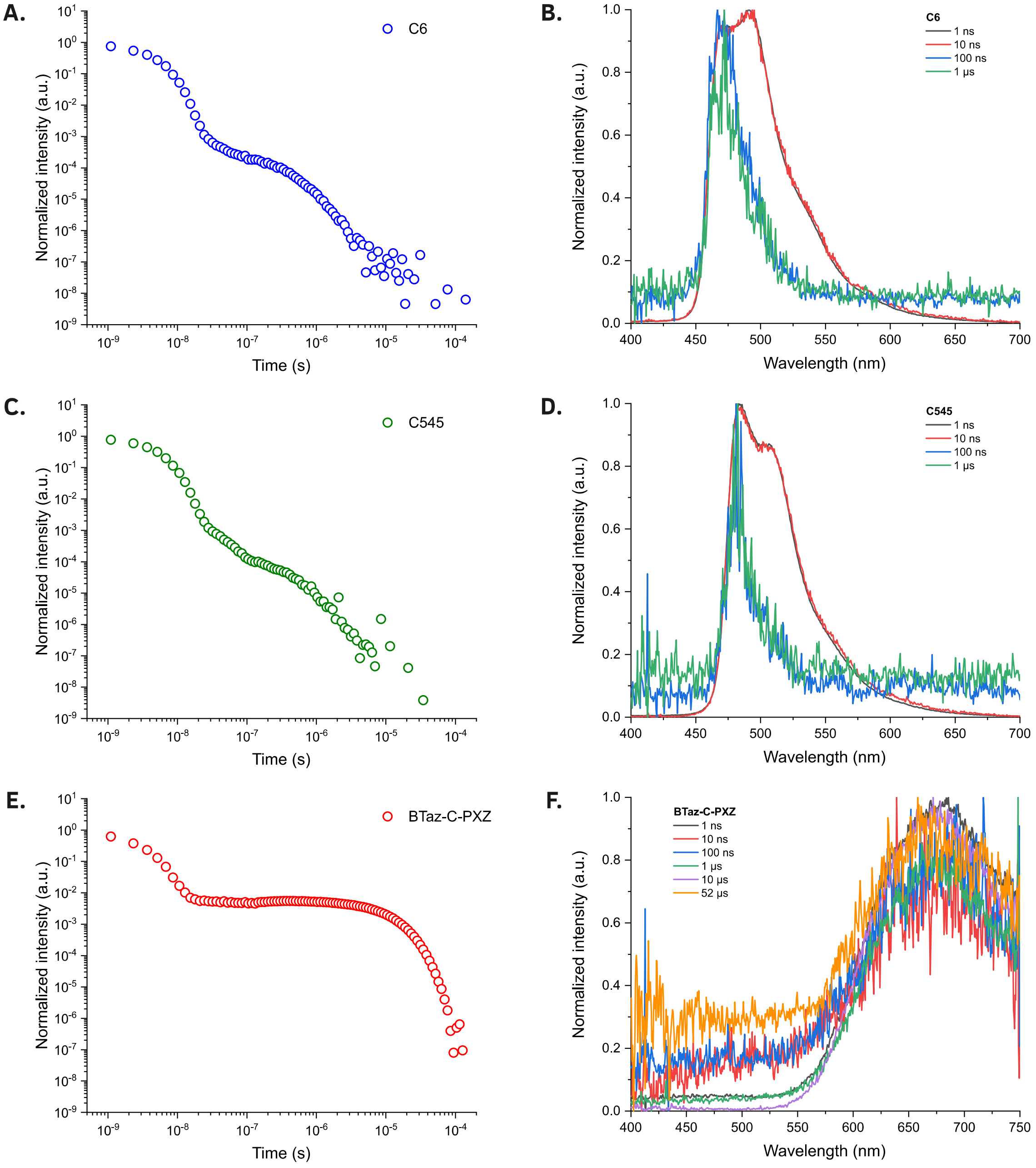

Figure S8: *Decay of the total emission for **C6** (A), **C545** (C), and **BTaz-C-PXZ** (E) in toluene at room temperature. Detailed spectra of the emission at various times during the decay for **C6** (B), **C545** (D), and **BTaz-C-PXZ** (F) in toluene at room temperature.*

8. Calculations of the TICT state of C6

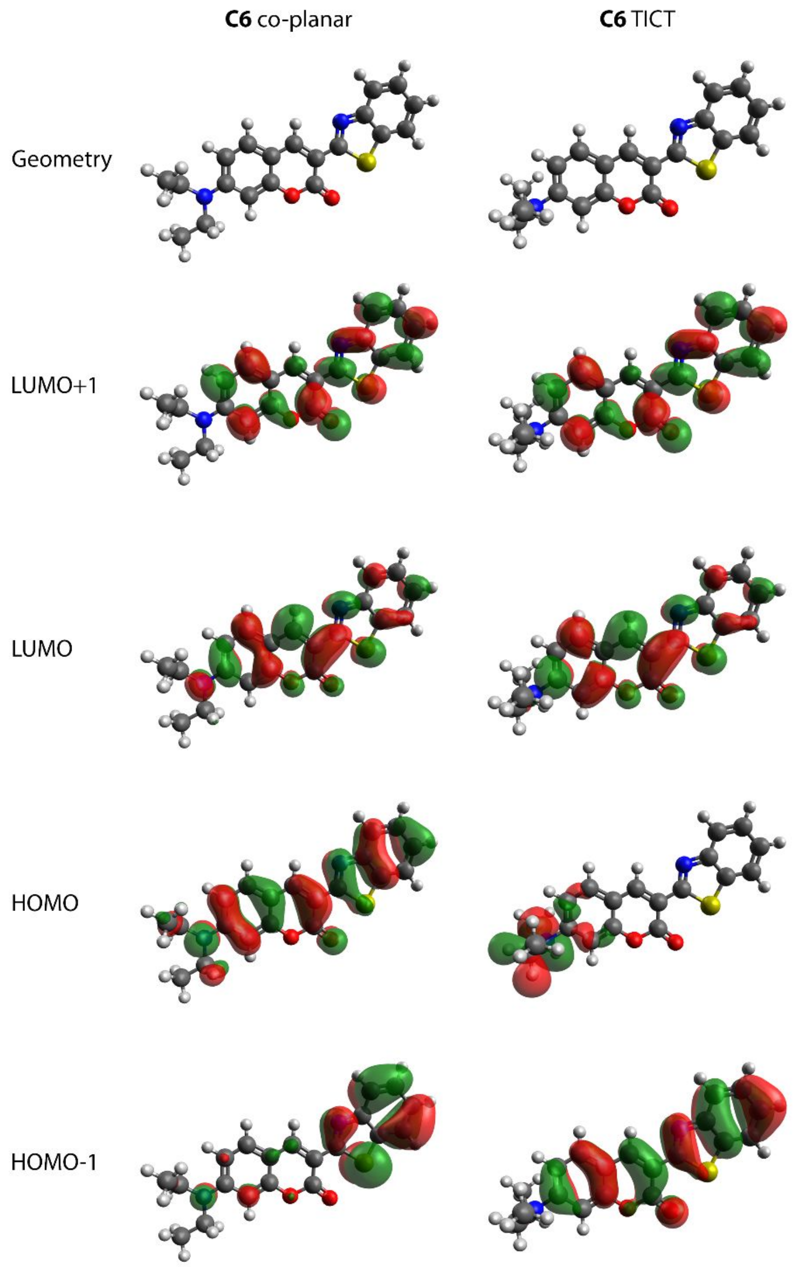

Figure S9: *Orbital spatial distributions for* ***C6****, comparing the planar ground state with the excited TICT state with an enforced 90° dihedral angle.*

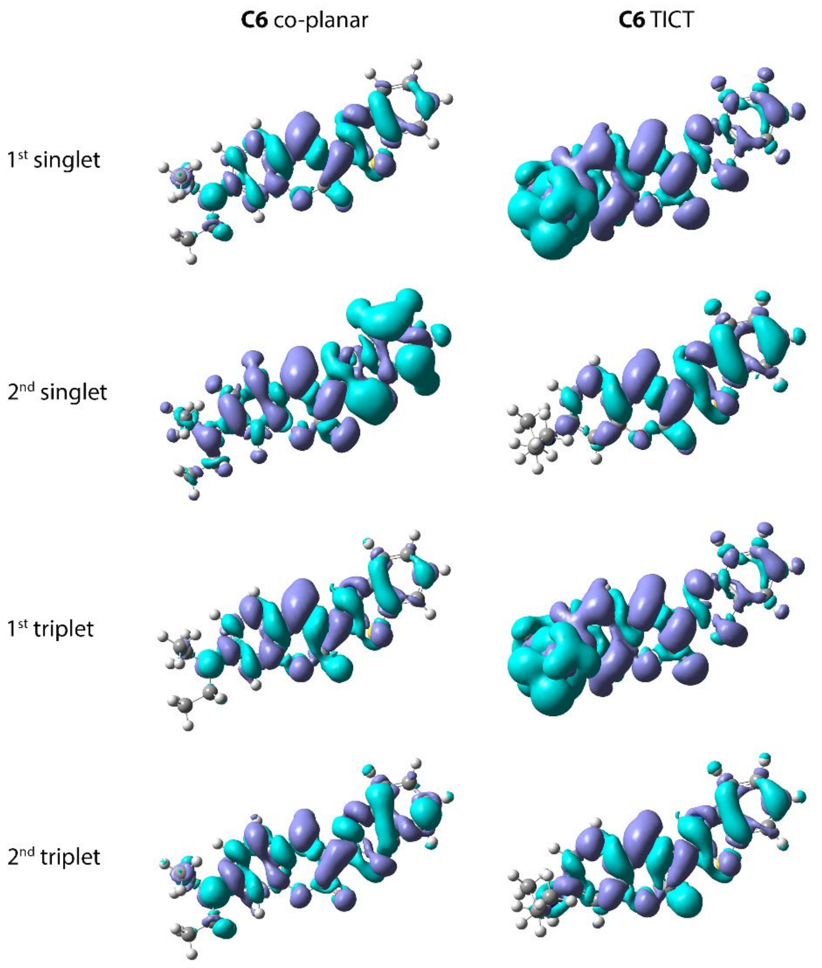

Figure S10: *Ground–excited state electron density differences for the singlet and triplet excited states of **C6** as calculated at the PCM(cyclohexane)/TDA/LC-BLYP(ω=0.17)/6-311G(d) level of approximation, comparing the planar ground state with the S_1 excited TICT state. Purple areas indicate increased electron density, while cyan areas point to decreased electron density (isosurface value = 0.0004 a.u. for all densities).*

9. NMR spectra

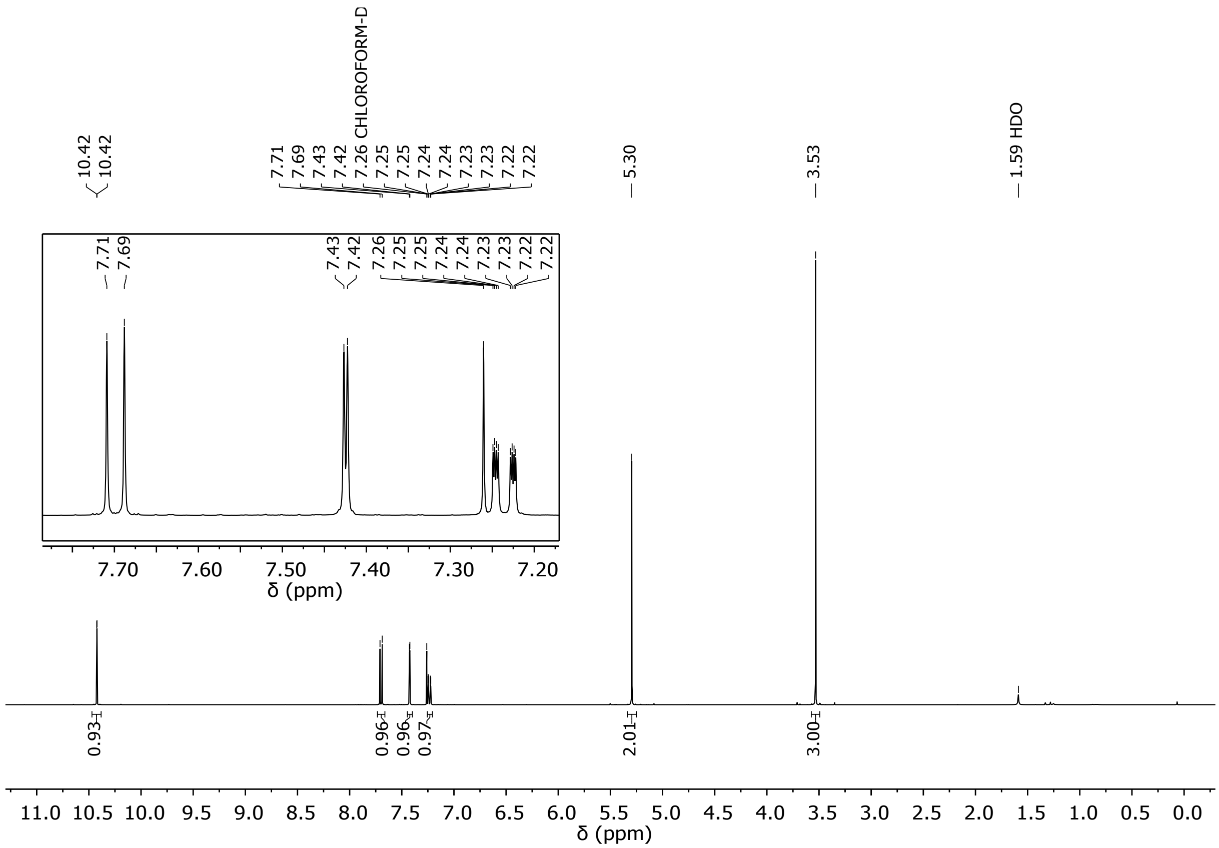

Figure S11: *^{1}H NMR spectrum of 4-bromo-2-(methoxymethoxy)benzaldehyde (**2**) in $CDCl_3$ (400 MHz).*

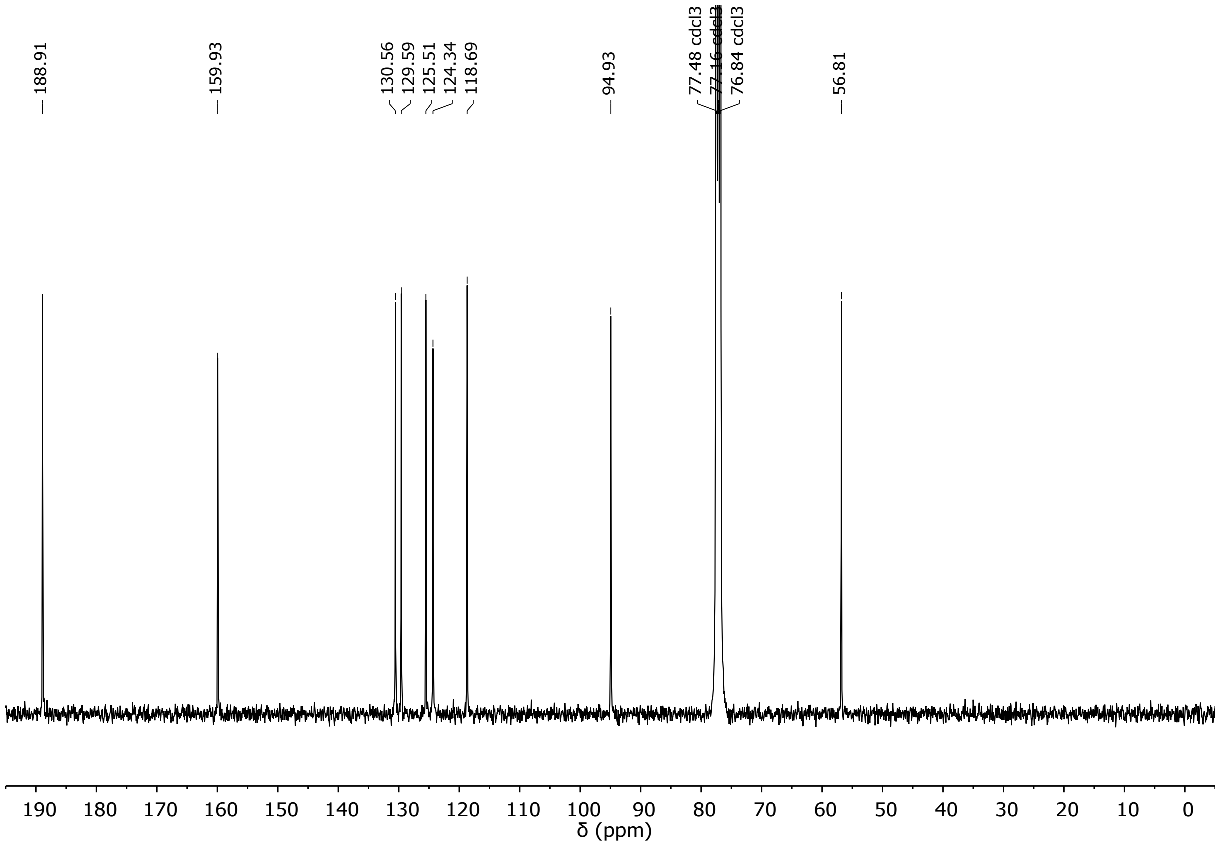

Figure S12: *^{13}C NMR spectrum of 4-bromo-2-(methoxymethoxy)benzaldehyde (**2**) in $CDCl_3$ (100 MHz).*

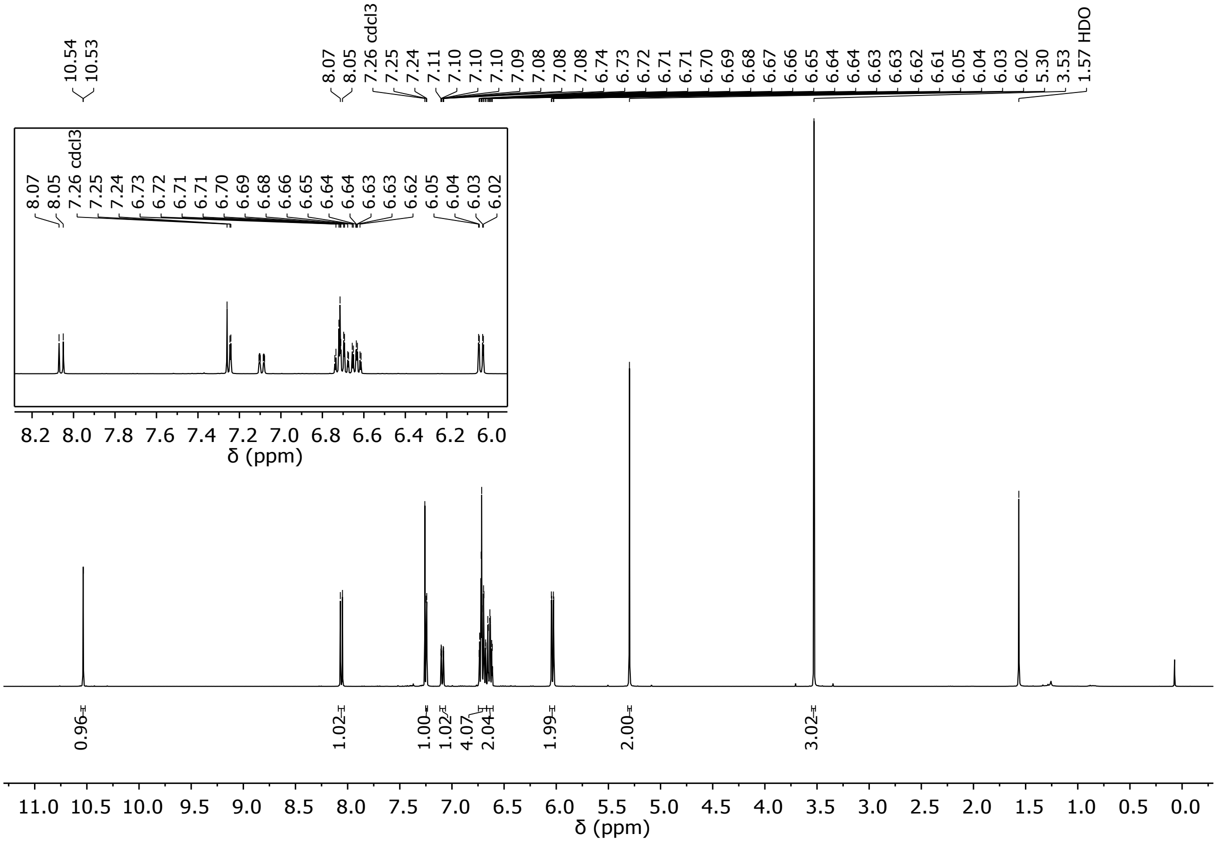

Figure S13: *^{1}H NMR spectrum of 2-(methoxymethoxy)-4-(10H-phenoxazin-10-yl)benzaldehyde (**3**) in $CDCl_3$ (400 MHz).*

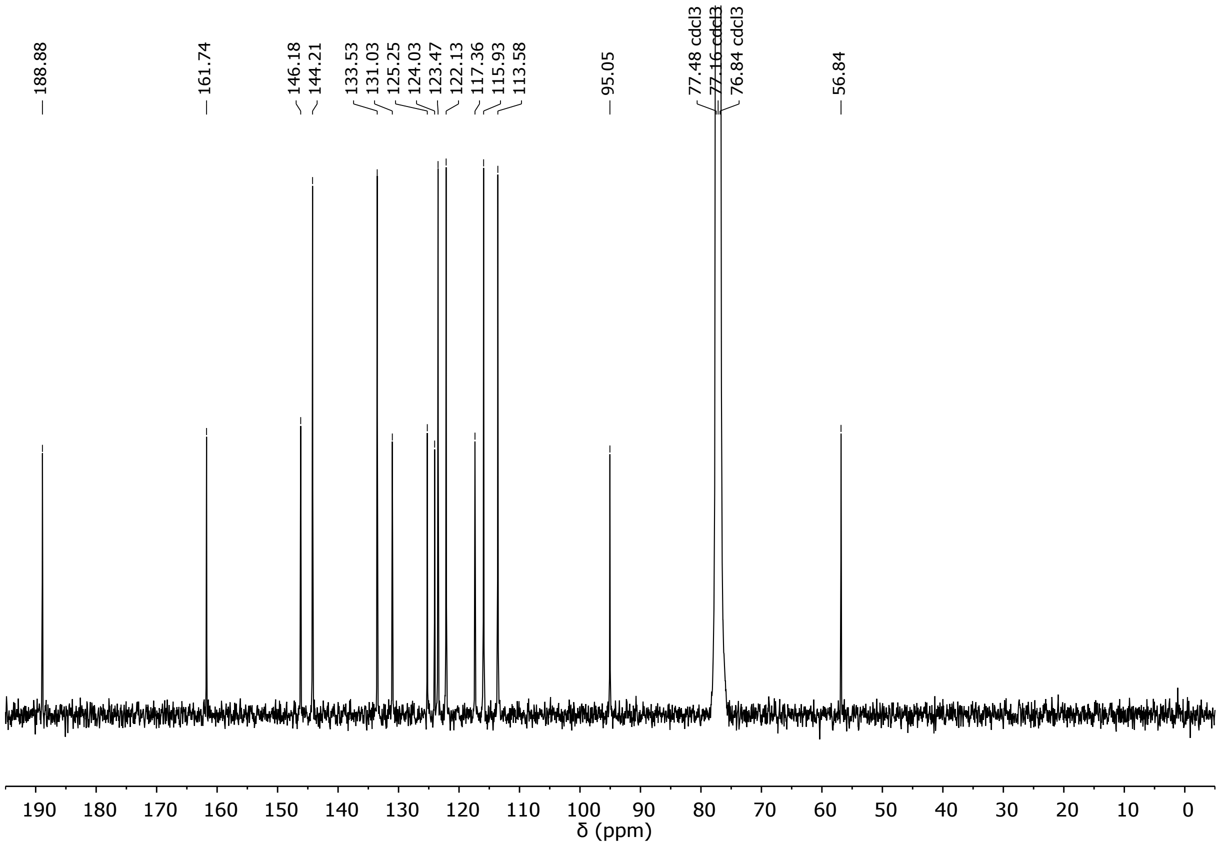

Figure S14: *^{13}C NMR spectrum of 2-(methoxymethoxy)-4-(10H-phenoxazin-10-yl)benzaldehyde (**3**) in $CDCl_3$ (100 MHz).*

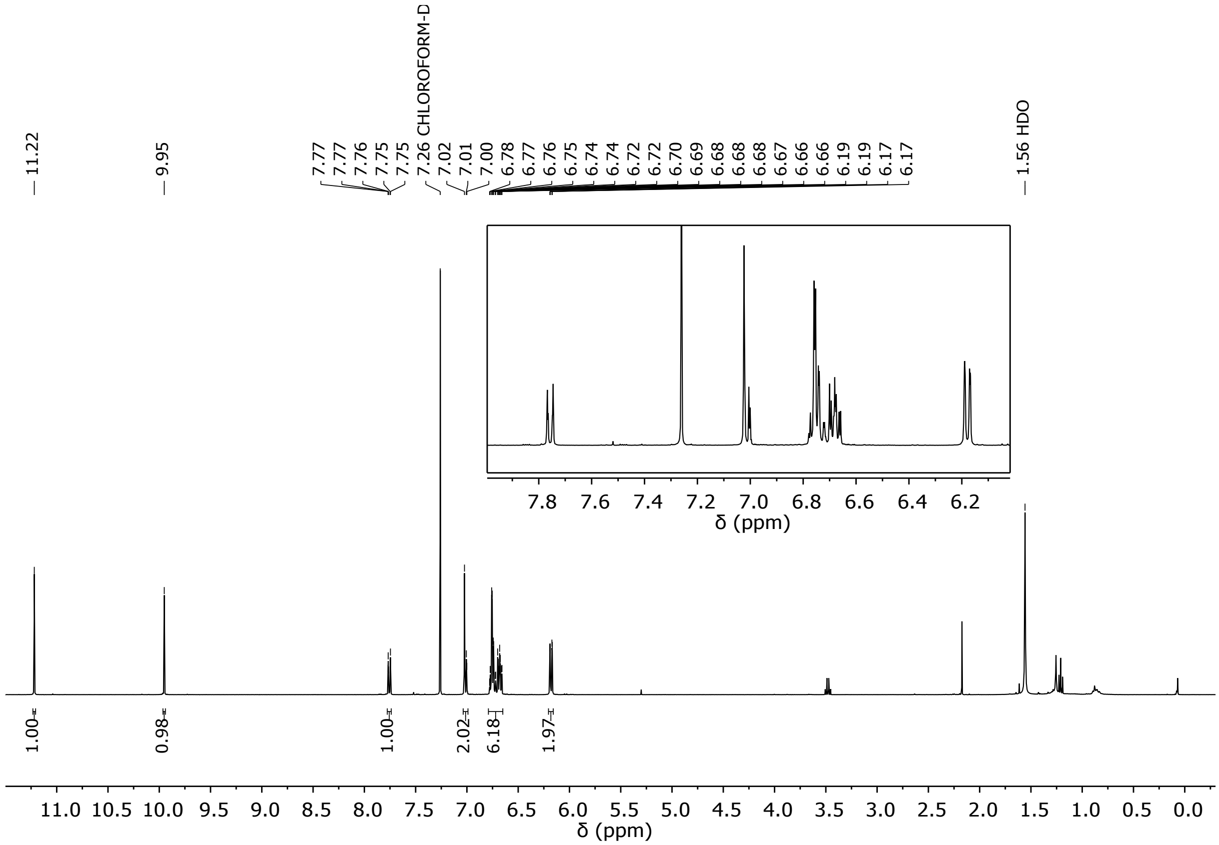

Figure S15: *^{1}H NMR spectrum of 2-hydroxy-4-(10H-phenoxazin-10-yl)benzaldehyde (**4**) in $CDCl_3$ (400 MHz).*

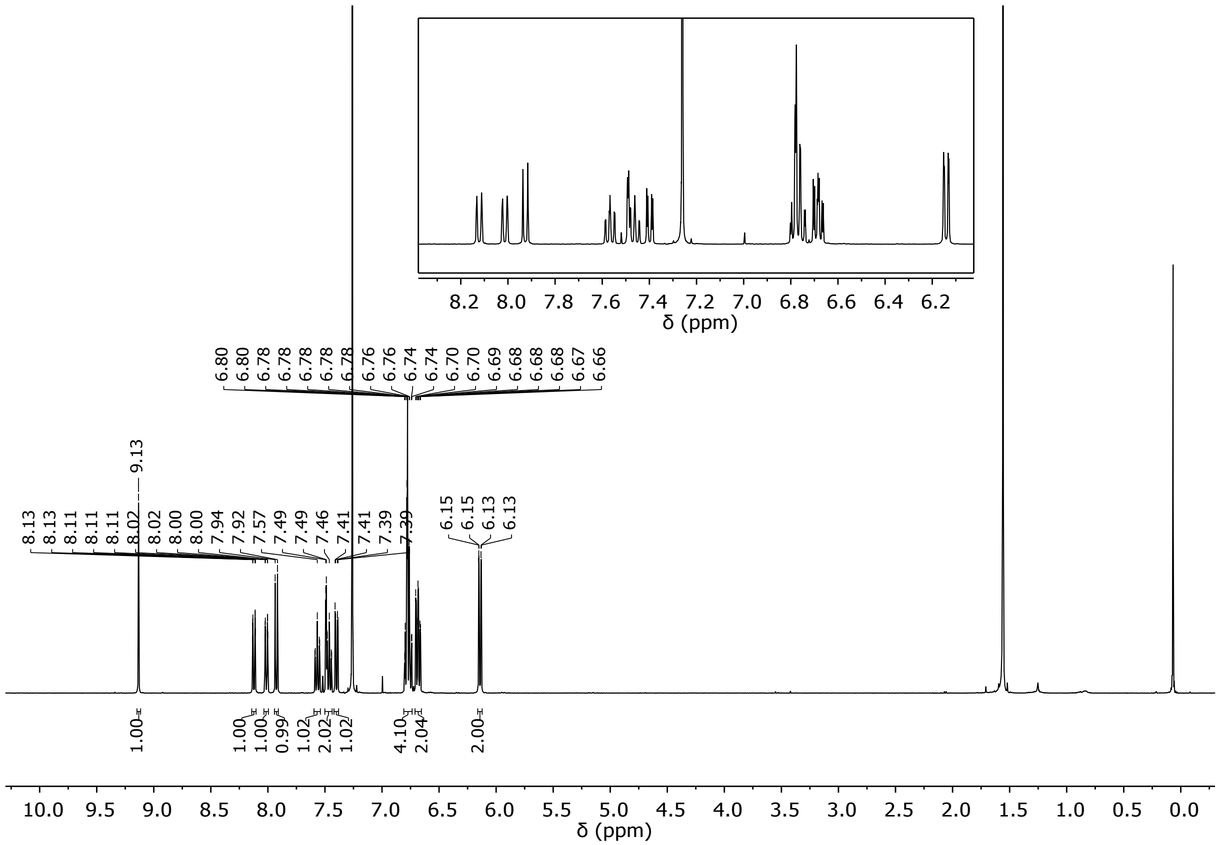

Figure S16: *^{1}H NMR spectrum of* ***BTaz-Coumarin-PXZ*** *in $CDCl_3$ (400 MHz).*

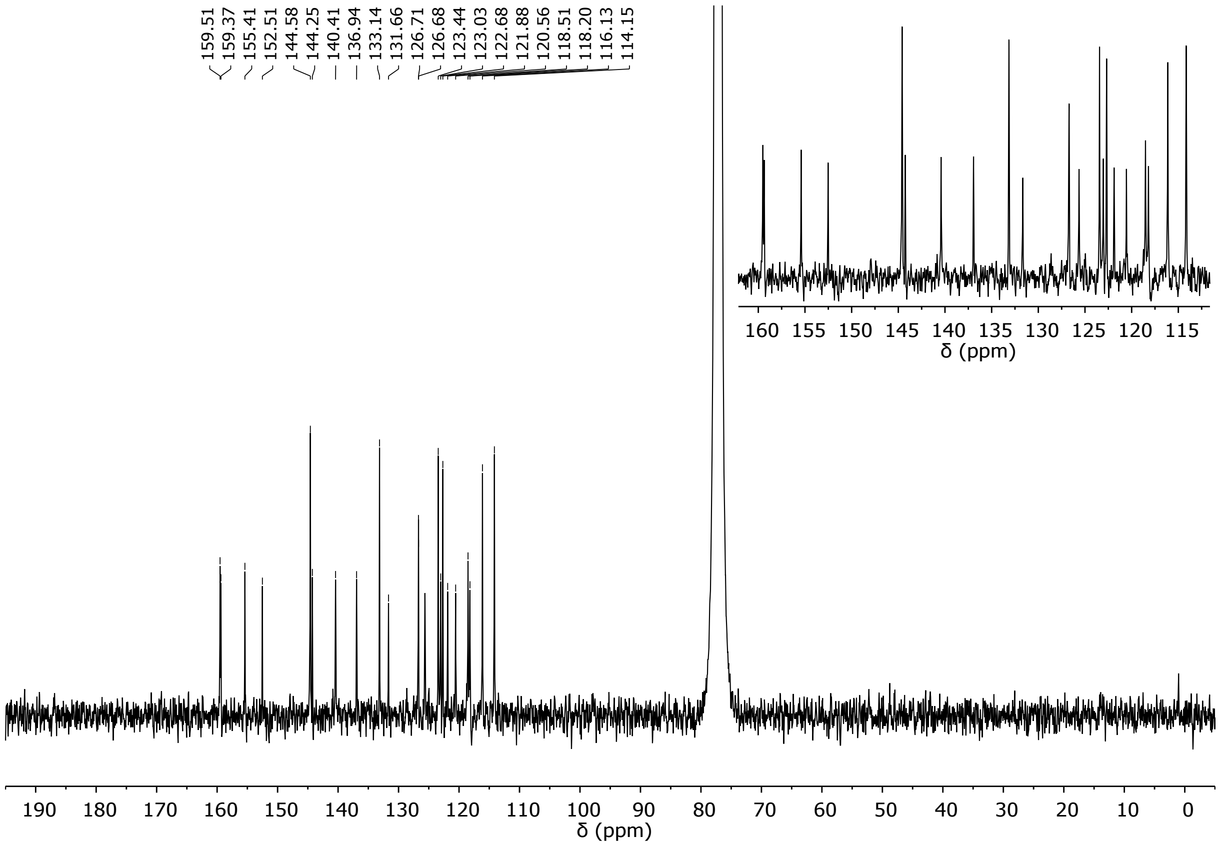

Figure S17: *^{13}C NMR spectrum of* ***BTaz-Coumarin-PXZ*** *in $CDCl_3$ (100 MHz).*

10. Coordinates of optimized geometries

C6

C	3.29785	-1.65365	-0.34523
C	3.87514	-0.35802	-0.25513
C	2.99735	0.72873	-0.11416
C	1.63106	0.52763	-0.10087
C	1.05799	-0.74441	-0.20736
C	1.93993	-1.82925	-0.32760
O	0.84958	1.62331	0.03256
C	-0.53094	1.57919	0.07010
C	-1.13920	0.26158	-0.04216
C	-0.35195	-0.84184	-0.17382
O	-1.11110	2.62123	0.19145
C	-2.58974	0.12399	-0.01137
N	-3.16966	-1.03102	-0.10677
N	5.23754	-0.17283	-0.31722
S	-3.67342	1.51361	0.15865
C	-4.53314	-0.91178	-0.05274
C	-5.01228	0.40294	0.09106
C	-5.44125	-1.97188	-0.12857
C	-6.79211	-1.70309	-0.06053
C	-7.25725	-0.38998	0.08245
C	-6.37601	0.67190	0.15937
C	6.12920	-1.29834	-0.07684
C	5.75631	1.17357	-0.09138
C	6.23394	-1.67563	1.38965
C	7.23415	1.34376	-0.36270
H	3.92732	-2.53133	-0.43557
H	3.33993	1.75014	-0.00212
H	1.52541	-2.83302	-0.40444
H	-0.83595	-1.81479	-0.25320
H	-5.06234	-2.98491	-0.23962
H	-7.50804	-2.51967	-0.11850
H	-8.32725	-0.20232	0.13369
H	-6.74051	1.69047	0.27036
H	5.80259	-2.15027	-0.68057
H	7.11400	-1.04378	-0.47592
H	5.21495	1.85361	-0.75999
H	5.52537	1.50572	0.93690
H	6.91779	-2.51877	1.53448z
H	6.60998	-0.83592	1.98634
H	5.25813	-1.95834	1.79949
H	7.48628	2.40561	-0.28418
H	7.87031	0.81078	0.35129
H	7.49947	1.01715	-1.37396

C545

C	-3.23382	1.58639	-0.13903
C	-3.76147	0.26857	-0.03326
C	-2.89211	-0.83723	0.03610
C	-1.52850	-0.60096	0.01636
C	-0.98230	0.68758	-0.06493
C	-1.87574	1.76721	-0.13822
O	-0.71315	-1.68085	0.09075
C	0.66717	-1.60952	0.08888
C	1.24179	-0.27795	0.00816
C	0.42350	0.81074	-0.06547
O	1.26921	-2.64515	0.15456
C	2.68785	-0.10620	0.00472

N	3.24085	1.06383	-0.06858
N	-5.11781	0.07943	-0.00445
S	3.80607	-1.47646	0.10288
C	4.60767	0.97316	-0.05257
C	5.11868	-0.33433	0.03791
C	5.49122	2.05450	-0.11752
C	6.84907	1.81362	-0.09098
C	7.34564	0.50768	-0.00043
C	6.48893	-0.57510	0.06447
C	-6.06655	1.17152	0.12739
C	-5.71579	-1.24358	-0.01700
C	-5.41692	2.44834	0.60938
C	-4.78475	-2.28252	-0.59639
C	-3.44464	-2.22817	0.11282
C	-4.18956	2.73663	-0.23528
H	-1.46984	2.77710	-0.20401
H	0.88386	1.79647	-0.12681
H	5.08837	3.06199	-0.18746
H	7.54560	2.64740	-0.14083
H	8.42036	0.34209	0.01903
H	6.87748	-1.58844	0.13463
H	-6.56940	1.34214	-0.84104
H	-6.85053	0.85337	0.83088
H	-6.64281	-1.18589	-0.60600
H	-6.01458	-1.52645	1.00851
H	-6.14480	3.26589	0.55958
H	-5.12158	2.34041	1.66219
H	-5.24357	-3.27334	-0.50616
H	-4.64242	-2.08931	-1.66867
H	-2.73460	-2.93792	-0.32231
H	-3.56708	-2.53196	1.16449
H	-3.69704	3.66482	0.07770
H	-4.50048	2.88768	-1.28171

BTaz-C-PXZ

C	1.69353	0.75372	-1.46093
C	2.22519	0.16254	-0.30882
C	1.38975	-0.34254	0.67413
C	0.01688	-0.26039	0.49771
C	-0.53705	0.33202	-0.64204
C	0.32923	0.84013	-1.62026
O	-0.77293	-0.76435	1.47255
C	-2.14975	-0.73572	1.42893
C	-2.74820	-0.12129	0.24485
C	-1.95725	0.38191	-0.73466
O	-2.74645	-1.21078	2.35081
C	-4.20151	-0.05079	0.12018
N	-4.76486	0.49151	-0.91261
N	3.63030	0.07874	-0.14688
S	-5.30073	-0.68928	1.34805
C	-6.12958	0.44649	-0.81676
C	-6.62511	-0.16283	0.35061
C	-7.02346	0.94868	-1.76788
C	-8.37714	0.83402	-1.53749
C	-8.85914	0.22501	-0.37112
C	-7.99349	-0.27745	0.58080
C	4.37815	1.23520	0.12937
C	5.77077	1.13611	0.21696
O	6.42703	-0.05438	0.05272
C	5.69180	-1.17598	-0.22669
C	4.29765	-1.14329	-0.33850
C	6.38990	-2.35218	-0.40683

C	5.71706	-3.52893	-0.71182
C	4.34063	-3.50798	-0.84176
C	3.63649	-2.32328	-0.66007
C	3.79183	2.47657	0.35056
C	4.57160	3.59396	0.62539
C	5.94855	3.48482	0.69109
C	6.54410	2.24570	0.48928
H	2.37507	1.13969	-2.21487
H	1.79518	-0.80120	1.57228
H	-0.09688	1.29658	-2.51141
H	-2.43179	0.83304	-1.60512
H	-6.63097	1.41788	-2.66651
H	-9.08354	1.22013	-2.26833
H	-9.93200	0.14652	-0.21215
H	-8.37208	-0.74898	1.48440
H	7.47180	-2.31912	-0.30585
H	6.27421	-4.45131	-0.85043
H	3.79650	-4.41567	-1.08936
H	2.55607	-2.31662	-0.77480
H	2.70969	2.57013	0.31470
H	4.08609	4.55184	0.79294
H	6.56497	4.35363	0.90481
H	7.62150	2.11111	0.54320

C6 TICT state

C	3.49264	-1.55135	0.13411
C	3.97199	-0.23871	-0.01807
C	3.09460	0.86945	-0.11262
C	1.73732	0.63007	-0.07896
C	1.19879	-0.68913	0.05740
C	2.13467	-1.77175	0.16639
O	0.91569	1.71304	-0.18786
C	-0.50291	1.60111	-0.17303
C	-1.03795	0.28601	-0.03875
C	-0.18517	-0.82711	0.07420
O	-1.10804	2.65549	-0.27817
C	-2.47014	0.10356	-0.02101
N	-3.04566	-1.06638	0.09612
N	5.37514	0.02172	-0.03019
S	-3.59237	1.49345	-0.16263
C	-4.42177	-0.95820	0.08285
C	-4.92480	0.35709	-0.05030
C	-5.32039	-2.02999	0.18833
C	-6.68607	-1.77695	0.16022
C	-7.17333	-0.46636	0.02792
C	-6.29754	0.61037	-0.07832
C	6.08271	0.07697	-1.30463
C	6.11557	0.33855	1.18775
C	6.07620	-1.28320	-1.99940
C	5.34408	0.08983	2.46565
H	4.19299	-2.38546	0.22275
H	3.45684	1.89334	-0.21858
H	1.74661	-2.78683	0.27349
H	-0.64754	-1.81177	0.17627
H	-4.92583	-3.04308	0.29063
H	-7.39142	-2.60728	0.24199
H	-8.25085	-0.28828	0.00788
H	-6.67742	1.62938	-0.18151
H	5.54992	0.82078	-1.91710
H	7.10434	0.43580	-1.11906
H	7.05471	-0.23788	1.13870
H	6.41191	1.39885	1.08538

H	6.61471	-1.19630	-2.95043
H	6.58303	-2.04309	-1.38819
H	5.05285	-1.61753	-2.20676
H	5.97632	0.36187	3.32101
H	4.42708	0.69055	2.50617
H	5.06111	-0.96546	2.56768
